# Vicinal Diol Sesquiterpenes from *Cinnamomum migao* with Neuroprotective Effects in PC12 Cells

**DOI:** 10.3390/ijms252312693

**Published:** 2024-11-26

**Authors:** Lang Zhou, Faju Chen, Lishou Yang, Mei Peng, Xiong Pan, Huayong Lou, Juan Yang, Xiaosheng Yang, Qiji Li

**Affiliations:** 1 State Key Laboratory of Functions and Applications of Medicinal Plants, Guizhou Medical University, Guiyang 550014, China; zhoulang913245@163.com (L.Z.); chenfaju202212@126.com (F.C.); 18798853904@163.com (L.Y.); pengmei520@163.com (M.P.); 18085124495@163.com (X.P.); loouhy@126.com (H.L.); yangxz2002@126.com (J.Y.); 2 Natural Products Research Center of Guizhou Province, Guiyang 550014, China

**Keywords:** *Cinnamomum migao* H. W. Li, Lauraceae, sesquiterpenoids, neuroprotective activity

## Abstract

In the ongoing search for new vicinal diol natural products, four new (Migaones A–D, **1**–**4**) and four known (**5**–**8**) vicinal diol sesquiterpenoids were isolated from the branches and leaves of *Cinnamomum migao*. Their structures were unequivocally determined by comprehensive spectroscopic analyses (HRESIMS, 1D, and 2D NMR), single-crystal X-ray diffraction, electronic circular dichroism calculations, and comparison with existing literature data. All compounds isolated from *C. migao* possess vicinal diol structural units except compound **2**. The newly isolated compounds (**1**–**4**) were evaluated for their neuroprotective activity using the PC12 cell injury model induced by *N*-methyl-daspartate acid (NMDA) and compounds **1**–**2** showing moderate neuroprotective activity against NMDA-induced neurotoxicity. Furthermore, molecular docking studies indicated that the most active compound **2** binds to the active site of the NMDA receptor via hydrogen bonds and hydrophobic interactions.

## 1. Introduction

Vicinal diol compounds are essential constituents of natural products, playing significant roles in the prevention and treatment of various diseases. Notable examples include the adrenergic drug epinephrine [1], antibiotic drug erythromycin [2], and the heart failure medication dapaglifozin [3]. In addition, vicinal diols are also crucial as synthetic intermediates. Thus, it is crucial to persistently investigate active vicinal diol natural products from plants.

*Cinnamomum migao* H. W. Li is an endemic medicinal plant, which is only distributed in the Guizhou, Yunnan, and Guangxi provinces of China [4]. Commonly known as Da-guo-mu-jiang-zi, it is a traditional remedy among Miao and Buyi ethnic minorities that is primarily used for treating arrhythmias, cerebrovascular diseases, and other ailments [5,6]. Additionally, *C. migao* is a key ingredient of Xinwei Zhitong Capsules and Liqi Huoxue Dropping Pills.

Sesquiterpenoids have demonstrated significant neuroprotective effects. For instance, atractylenolide III protects PC12 cells from corticosterone-induced injury [7], japonipene A-C exhibit neuroprotective effects against CoCl_2_-induced neuronal cell death in SH-SY5Y cells [8], flammuterpenols A–D protect against 6-hydroxydopamine-induced cell death in SH-SY5Y cells [9], and inderaggrols A−F show neuroprotective effects against erastin-induced ferroptosis in HT-22 cells [10]. Overall, sesquiterpenoids have considerable potential as neuroprotective agents.

**Figure 1:**
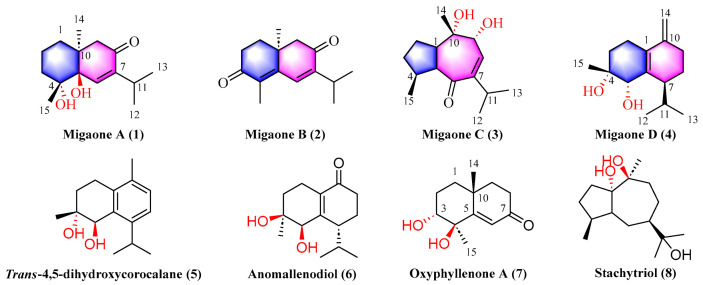
Structures of compounds **1**–**8**.

Previous phytochemical investigations of *C. migao* have revealed that the plant is rich in guaiane-type sesquiterpenes, which exhibit notable anti-inflammatory activity [11,12]. Our earlier research identified a novel artemisinin-like sesquiterpenoid featuring an unprecedented tetracyclic 6/6/7/5 ring system, which demonstrated neuroprotective activity [13]. In this study, we report the isolation of four previously undescribed (**1**−**4**) and four known (**5**−**8**) sesquiterpenoids (Figure 1) from the branches and leaves of *C. migao*. Compounds **1**–**2** exhibited moderate neuroprotective activity against NMDA-induced neurotoxicity at 30 μM. Here, we report the isolation, structure elucidation, and neuroprotective activity of these undescribed compounds. This finding enriched the chemical composition of *C. migao* and provided a reference for the development and utilization of the plant resource in the future.

## 2. Results

### 2.1. Structure Elucidation

Migaone A (**1**) was isolated as a colorless needle crystal. The molecular formula, C_15_H_24_O_3_, was established by HR-ESI-MS ([M+Na]^+^ 275.1614, calcd for 275.1623), corresponding to four indices of hydrogen deficiency (IHDs). The ^1^H NMR spectrum (Table 1) of compound **1** revealed an olefinic proton [*δ*_H_ 6.87 (1H, s, H-6)] and four methyl groups [*δ*_H_ 1.07 (3H, d, *J* = 6.9 Hz, H_3_-12), 1.05 (3H, d, *J* = 6.9 Hz, H_3_-13), 1.23 (3H, s, H_3_-14), 1.37 (3H, s, H_3_-15). The ^13^C NMR and HSQC spectrum exhibited 15 carbon resonances, which were assigned as four methyls (*δ*_C_ 21.5, 22.1, 23.6, 26.7), four methylenes (*δ*_C_ 17.4, 33.0, 36.3, 51.2), two methines (*δ*_C_ 26.4, and an olefinic carbon *δ*_C_ 139.7), and five quaternary carbons (*δ*_C_ 41.3, two oxygenated carbons *δ*_C_ 74.4, 73.8, an olefinic carbon *δ*_C_ 148.5, and a ketone carbonyl *δ*_C_ 199.8). Taking into account the double bond and keto carbonyl, the two remaining IHDs suggest that compound **1** is a dicyclic sesquiterpene.

**Figure 2:**
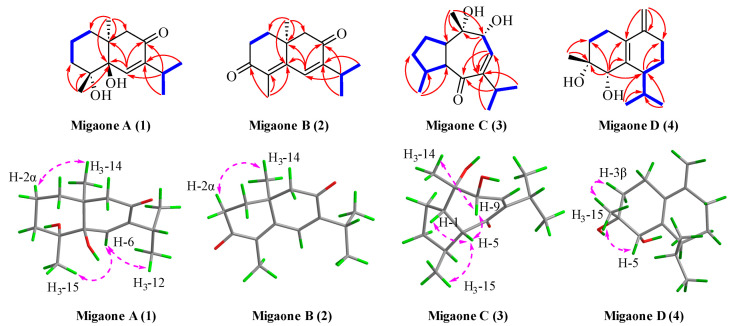
Key 2D NMR correlations of **1**–**4**.

The planar structure of compound **1** was accomplished by 2D NMR spectroscopic analysis. The ^1^H−^1^H COSY correlations of H_2_-1/H_2_-2/H_2_-3 and H_3_-12/H-11/H_3_-13 revealed two key spin systems (Figure 2). The HMBC correlations (Figure 2) from H_3_-12 (*δ*_H_ 1.05)/H_3_-13 (*δ*_H_ 1.07) to C-7 (*δ*_C_ 148.5), C-11 (*δ*_C_ 26.4), from H-11 (*δ*_H_ 2.90) to C-6 (*δ*_C_ 139.7), C-7 (*δ*_C_ 148.5), C-8 (*δ*_C_ 199.8), C-12 (*δ*_C_ 22.1), C-13 (*δ*_C_ 21.5), from H_3_-14 (*δ*_H_ 1.23) to C-1 (*δ*_C_ 33.0), C-5 (*δ*_C_ 73.8), C-9 (*δ*_C_ 51.2), C-10 (*δ*_C_ 41.3), and from H_3_-15 (*δ*_H_ 1.37) to C-3 (*δ*_C_ 36.3), C-4 (*δ*_C_ 74.4), C-5 (*δ*_C_ 73.8) provided evidence for the presence of hydroxyl groups at C-4 and C-5, a double bond between C-6 and C-7, and a ketone carbonyl at C-8. Therefore, the planar structure of 1 was determined to be an eudesmane-type sesquiterpenoid with a 6/6 ring system.

**Figure 3:**
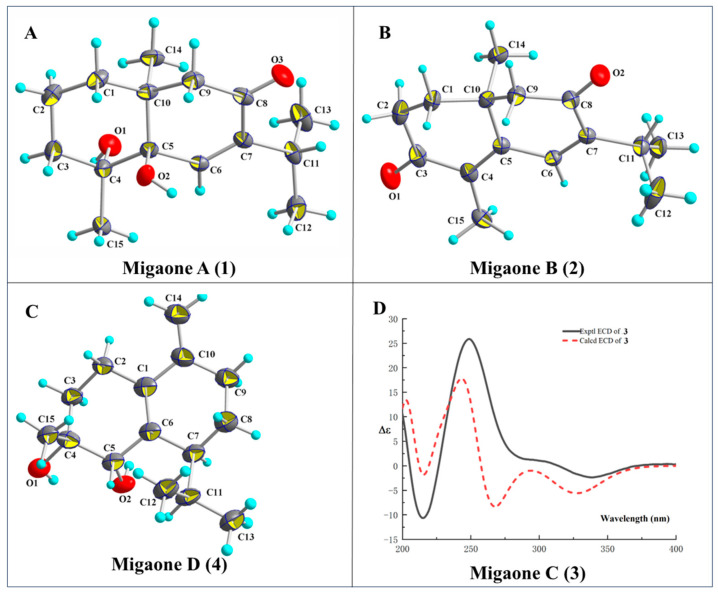
(**A**–**C**) X-ray of compounds of **1**, **2**, **4**. (**D**) Experimental and calculated ECD of **3**.

The relative configuration of compound **1** was assigned based on the analysis of Nuclear Overhauser Effect Spectroscopy (NOESY) cross-peaks involving H_3_-14/H-2*α*, H_3_-15/H-6/H_3_-12 (Figure 2). These data indicated that H_3_-14 is *α*-oriented and H_3_-15 is *β*-oriented. Finally, a single-crystal X-ray diffraction study was performed (CCDC number: 2377460) (Figure 3), confirming the above structural assignments and determining the absolute configuration as (4*R*, 5*R*, 10*S*), which was named Migaone A.

Migaone B (**2**) was obtained as a colorless needle crystal. Its molecular formula was determined to be C_15_H_20_O_2_ from HR-ESI-MS at *m*/*z* 233.1533 [M+H]^+^ (calcd for 233.1536). which corresponds to six IHDs. The ^1^H and HSQC spectrum revealed four methyl groups at *δ*_H_ 1.11 (3H, d, *J* = 6.9 Hz, H_3_-12), 1.14 (3H, d, *J* = 6.9 Hz, H_3_-13), 1.25 (3H, s, H_3_-14), and 1.97 (3H, s, H_3_-15) and an olefinic proton [*δ*_H_ 7.16 (1H, s, H-6)]. The ^13^C-NMR and HSQC spectra of compound **2** suggested the presence of 15 carbon resonances, including four methyls (*δ*_C_ 11.2, 21.8, 22.0, 23.9), three methylenes (*δ*_C_ 33.7, 35.9, 53.0), two methines (*δ*_C_ 27.2, a double carbon signal *δ*_C_ 133.6), and six quaternary carbons (*δ*_C_ 37.5, three double-bond carbons *δ*_C_ 133.3, 148.8, 152.1, two ketone carbonyl carbons *δ*_C_ 197.9, 198.6), accounting for four IHDs. Accordingly, the remaining two IHDs indicated that compound **2** has a dicyclic ring system.

In the 2D NMR data, two isolated spin systems were identified from the ^1^H−^1^H COSY spectrum, namely, H_2_-1/H_2_-2 and H_3_-12/H-11/H_3_-13 (Figure 2). The HMBC spectrum (Figure 2) revealed the following correlations, from H-6 (*δ*_H_ 7.16) to C-4 (*δ*_C_ 133.3), C-7 (*δ*_C_ 148.8), C-8 (*δ*_C_ 197.9) and C-10 (*δ*_C_ 37.5), from H-11 (*δ*_H_ 3.02) to C-6 (*δ*_C_ 133.6), C-7 (*δ*_C_ 148.8), C-8 (*δ*_C_ 197.9), C-12 (*δ*_C_ 22.0) and C-13 (*δ*_C_ 21.8), from H_3_-12 (*δ*_H_ 1.11)/H_3_-13 (*δ*_H_ 1.14) to C-7 (*δ*_C_ 148.8) and C-11 (*δ*_C_ 27.2), from H_3_-14 (*δ*_H_ 1.25) to C-1 (*δ*_C_ 35.9), C-5 (*δ*_C_ 152.1), C-9 (*δ*_C_ 53.0) and C-10 (*δ*_C_ 37.5), and from H_3_-15 (*δ*_H_ 1.97) to C-3 (*δ*_C_ 198.6), C-4 (*δ*_C_ 133.3), and C-5 (*δ*_C_ 152.1). These correlations suggested the presence of two double bonds at *Δ*^4/5^ and *Δ*^6/7^ and two ketone carbonyls located at C-3 and C-8.

The NOESY correlations between H_3_-14 and H-2*α* indicated that these protons were oriented on the same side (*α*-configurations). Its absolute configuration was confirmed to be (10 *S*) by X-ray diffraction analysis (CCDC number: 2377461), as shown in Figure 3, and it was named Migaone B.

Migaone C (**3**) was obtained as a colorless oil. Its HR-ESI-MS peak at *m*/*z* 275.1614 ([M+Na]^+^, calcd for 275.1617) and the ^13^C NMR data suggested a molecular formula of C_15_H_24_O_3_, indicating four IHDs. Analysis of its 1D and 2D NMR data suggested that compound **3** was structurally similar to (4*R*,10*R*)-9,10-dihydroxy-7-isopropyl-4,10-dimethyl-1,3,4,5,9,10-hexahydroazulen-6(1H)-one, as reported in the literature [14], with the exception of configurations differences. The relative configuration of **3** was established based on the NOESY data (Figure 2). The NOE correlations of H-9/H_3_-14 and H_3_-15/H-5/H-1 suggested that H-1, H-5, H-9 and H_3_-14 were arbitrarily assigned as *β*-oriented, while the correlations of H-4/H-2*α* indicated that H-4 was *α*-oriented. Finally, the absolute configuration of **3** (1*S*, 4*S*, 5*R*, 9*R*, 10*S*) was established through electronic circular dichroism (ECD) calculations with time-dependent density functional theory (TD-DFT), which were consistent with experimental data (Figure 3), and named Migaone C.

Migaone D (**4**) was obtained as a colorless needle crystal. Its molecular formula, C_15_H_24_O_2_, corresponding to four IHDs, was determined by positive HR-ESI-MS ([M+Na]^+^ 259.1664, calcd for 259.1669). The ^1^H and ^13^C NMR spectra of **4** resembled those of (4*R*, 5*R*)-muurol-1(6),10(14)-diene-4,5-diol [15] except for the configuration of the hydroxyl group at C-4 position; this was confirmed by the NOESY correlations between H-3*β*/H_3_-15/H-5, which indicated that the protons were oriented on the same side (*β*-configurations), and 4-OH was assigned as *α*-orientations. The absolute configuration of **4** was determined to be (4*R*, 5*S*, 7*S*) through X-ray diffraction analysis (CCDC number: 2377462), as shown in Figure 3, and it was named Migaone D.

The known compounds were identified based on their spectroscopic data analysis and comparisons with previously reported compounds in the literature (Figure 1), which were identified as *trans*-4,5-dihydroxycorocalane (**5**) [16], anomallenodiol (**6**) [17], oxyphyllenone A (**7**) [18], and stachytriol (**8**) [19].

### 2.2. Neuroprotective Activity

**Figure 4:**
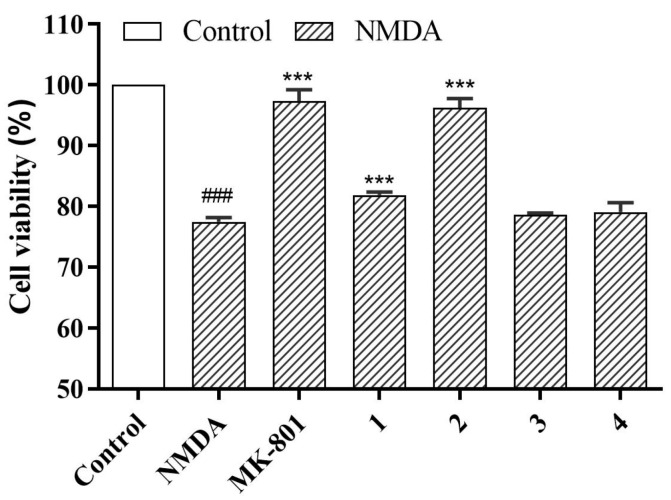
The neuroprotective effects of compounds **1**–**4** against NMDA-induced injury in PC12 cells (NMDA: 2 mM; compounds **1**–**4**: 30 μM; data are expressed as means ± SD, n = 3). ^###^ *p* < 0.001 vs. control group, *** *p* < 0.001 vs. NMDA group.

Pheochromocytoma (PC12) cells, derived from rat pheochromocytoma tumors, are widely used as neuronal cell lines in neurobiological studies [20]; they have the characteristics of nerve cells and are commonly employed in neuroprotective research. Previously, we isolated several undescribed guaiane-type sesquiterpenes with neuroprotective activity from the fruits of *C. migao* [9] as part of our ongoing exploration for structurally diverse sesquiterpenes with neuroprotective effects from *C. migao*. Compounds **1**–**4** were evaluated for their neuroprotective activity against NMDA-induced neurotoxicity in PC12 cells. Dizocilpine (MK801) was used as a positive control. The neuroprotective effects of compounds **1**–**4** are summarized in Figure 4. Compared to the model group (77.47 ± 0.68%), compounds **1** (81.85 ± 0.50%), **2** (96.21 ± 1.57%), and MK801 (97.33 ± 1.87%) demonstrated neuroprotective effects at 30 μM.

### 2.3. Molecular Docking Study

**Figure 5:**
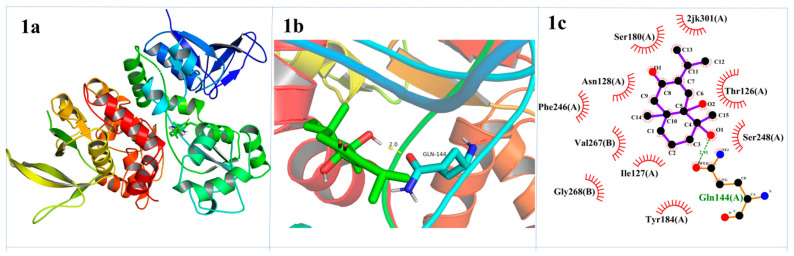

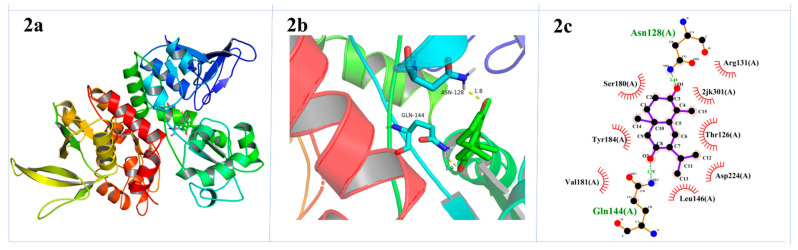
Molecular docking interaction of compounds **1**–**2** with NMDAR (PDB: 4NF4). Binding pose (**a**), detailed 3D (**b**) and 2D (**c**) protein interactions in the active site region of the molecules.

Molecular docking was employed to investigate the interaction between the active compounds and the NMDA receptor (NMDAR). Specifically, compounds **1**–**2** were docked into the glycine binding site of NMDAR (PDB ID: 4NF4) [21]. The docking results indicated that compounds **1**−**2** exhibited a favorable fit within the active pocket of NMDAR glycine antagonists. The calculated free energy change for compound **1** in its lowest energy conformation during molecular docking was −19.9995 kJ/mol. To facilitate a detailed analysis of the interactions between the ligand and NMDAR, Pymol software version 2.4 was used for visualization. Compound **1** established hydrogen bonds with amino acid residues GLN144 in the active pocket of NMDAR and also formed hydrophobic interactions with amino acid residues THR126, ASN128, GLN144, TYR184, and VAL267. The docking score for compound **2** was −24.2672 kJ/mol. Compound **2** also formed hydrogen bonds with GLN144 and ASN128. Additionally, it formed hydrophobic interactions with THR126, GLN144, LEU146, VAL181, and TYR184. The hydrogen bonding and hydrophobic interaction were key factors contributing to the binding affinity of these compounds to NMDAR. The results are shown in Figure 5.

## 3. Materials and Methods

### 3.1. General Experimental Procedures

X-ray crystallographic data were recorded on an XtaLAB AFC12 (RINC) using Cu Kα radiation. Optical rotation measurements were conducted at a wavelength of 589 nm using an MCP 500 apparatus at 25 °C. Melting points were determined on a MP30 melting point apparatus (Mettler Toledo, Zurich, Switzerland). Ultraviolet (UV) spectra were recorded on a Shimadzu-2600 spectrophotometer (Shimadzu, Tokyo, Japan). Electronic Circular Dichroism (ECD) spectra were obtained with a Chirascan spectrometer (Applied Photophysics Ltd., Leatherhead, UK). Infrared (IR) spectra (KBr) were measured using an IRAffinity-1 spectrometer; 1D and 2D spectra were collected on an AVANCE III-600 spectrometer with TMS as an internal standard (Bruker Corp, Bremen, Germany). HRESIMS was performed on an Agilent 6210 ESI/TOF mass spectrometer (Agilent Technologies Inc., Santa Clara, CA, USA). Analytical HPLC was conducted using an Agilent 1260 system equipped with a reversed-phase C18 column (5 μm, 250 mm × 4.60 mm, SHIMADZU shim-pack GIS, Shimadzu, Tokyo, Japan). Semi-preparative HPLC was performed using a Shimadzu LC-20A instrument equipped with a UV SPD-20A detector (Shimadzu, Tokyo, Japan) and a reversed-phase C18 column (5 μm, 250 mm × 10 mm, SHIMADZU shim-pack GIS, Shimadzu, Tokyo, Japan).

### 3.2. Plant Material

The branches and leaves of *Cinnamomum migao* H. W. Li were collected from Ceheng, Guizhou province, China, in October 2019. The plant was identified by Professor Qing-wen Sun. A voucher specimen (GZCH20191001) has been deposited at the Natural Products Research Center of Guizhou Province.

### 3.3. Extraction and Isolation

The branches and leaves of *C. migao* (21 kg) were extracted with 95% aqueous ethanol (4 × 30 L) under reflux three times (each time for 3 h) to yield a crude extract. The ethanol crude extract (1.0 kg) was suspended in water (10 L) and partitioned with petroleum ether (PE) and ethyl acetate (EtOAc), yielding PE (200 g) and EtOAc (300 g) layers, respectively.

The PE fraction (200 g) was subjected to silica gel column chromatography (CC) using a solvent gradient of PE–EtOAc (1:0 to 0:1, *v*/*v*), resulting in six fractions (Fr.A1–Fr.A6). Fr.A6 (300 mg) was further separated on an Rp-C18 column using a MeOH–H₂O gradient (60:40, 70:30, 80:20, 90:10, *v*/*v*), yielding four subfractions Fr.A6.1-Fr.A6.4. Fr.A6.1 (125 mg) was further purified by semi-preparative HPLC with CH_3_CN–H_2_O (65:35, *v*/*v*, 2 mL/min) to obtain compound **8** (65 mg, *t*_R_ = 25.3 min).

The EtOAc layers (300 g) were chromatographed over a silica gel column using a PE–EtOAc gradient solvent system (10:1 to 0:1, *v*/*v*), resulting in seven fractions (Fr.B1–Fr.B7). Fr.B2 (300 mg) was further separated on silica gel with a PE–EtOAc solvent system (20:1, *v*/*v*), yielding compound ***7*** (20 mg). Fr.B4 (4 g) was chromatographed on an Rp-C18 CC, using MeOH−H_2_O (40:60, 60:40, 70:30, *v*/*v*) as the eluent solvent to give three subfractions (Fr.B4.1-Fr.B4.3). Fr.B4.2 (220 mg) was purified by semi-preparative HPLC eluted with CH_3_CN–H_2_O (45:55, *v*/*v*, 2 mL/min) to obtain compounds **6** (11 mg, *t*_R_ = 25.4 min). Fr.B-5 (11 g) was separated on Rp-C18 gel with MeOH–H_2_O (40:60, 50:50, 60:40, 70:30, *v*/*v*) to yield four subfractions (Fr.B5.1-Fr.B5.4). Fr.B5.2 (231 mg) was separated by semi-preparative HPLC eluted with a MeOH–H_2_O (55:45, *v*/*v*, 2 mL/min) to afford **5** (16 mg, *t_R_* = 30.5 min). Fr.B5.2 (231 mg) was also separated by semi-preparative HPLC (MeOH–H_2_O, 70:30, *v*/*v*, 2 mL/min) to afford compound **4** (17 mg, *t_R_* = 23.6 min). Fr.B5.3 (60 mg) was separated on a silica gel column eluted with a PE−EtOAC gradient (3:1, *v*/*v*) to yield compound **1** (8 mg). Fr.B5.4 (72 mg) was purified by semi-preparative HPLC with MeOH–H_2_O (70:30, *v*/*v*, 2 mL/min) to afford **3** (4 mg, *t_R_* = 19.9 min) and **2** (4 mg, *t_R_* = 26.3 min).

Migaone A (**1**): colorless needle crystal (CHCl_3_–MeOH), [α${]}_{D}^{25}$ + 23.7 (*c* 0.11, MeOH); m.p 130.9 °C. UV (MeOH) *λ*_max_ (log *ε*): 235 (3.39) nm; IR (KBr) *υ*_max_ 3441, 2962, 2926, 2870, 1656, 1458, 1373, 1020, 995, 970, 948 cm^–1^. For ^1^H and ^13^C NMR see Table 1. HR-ESI-MS *m*/*z* 275.1614 [M+Na]^+^(calcd for [C_15_H_24_O_3_Na]^+^, 275.1623). Supporting information: Figures S1–S9.

Migaone B (**2**): colorless needle crystal (CHCl_3_–MeOH); [α${]}_{D}^{25}$ − 200 (*c* 0.12, MeOH). UV (MeOH) *λ*_max_ (log *ε*): 310 (3.58) nm. IR (KBr) *υ*_max_ 3446, 2960, 2926, 2872, 1660, 1300, 1199, 1022, 848 cm^–1^. ^1^H and ^13^C NMR, see Table 1. HR-ESI-MS *m*/*z* 233.1533 [M+H]^+^ (calculated for [C_15_H_21_O_2_]^+^, 233.1536). Supporting information: Figures S10–S18.

Migaone C (**3**): colorless oil. [α${]}_{D}^{25}$ + 31.9 (*c* 0.23, MeOH); UV (MeOH) *λ*_max_ (log *ε*): 201 (2.97), 241 (3.16) nm; ECD (MeOH) *λ (*Δ*ε*) 215 (−10.64), 249 (+25.87), 338 (−2.35) nm; IR (KBr) *υ*_max_ 3381, 2956, 2870, 1660, 1462, 1373, 1049, 669 cm^–1^. For ^1^H and ^13^C NMR, see Table 1. HR-ESI-MS *m*/*z* 275.1614 [M+Na]^+^ (calculated for [C_15_H_24_O_3_Na]^+^, 275.1617). Supporting information: Figures S19–S27.

Migaone D (**4**): colorless needle crystal (CHCl_3_–MeOH). [α${]}_{D}^{25}$ − 15.1 (*c* 0.20, MeOH). UV (MeOH) *λ*_max_ (log *ε*): 242 (3.49). IR (KBr) *υ*_max_ 3346, 2956, 2935, 1635, 1456, 1016, 667, 599, 555 cm^–1^. For ^1^H and ^13^C NMR, see Table 1. HR-ESI-MS *m*/*z* 259.1664 [M+Na]^+^ (calculated for [C_15_H_24_O_2_Na]^+^, 259.1669). Supporting information: Figures S28–S36.

*Trans*-4,5-dihydroxycorocalane (**5**): colorless oil, ESI-MS *m*/*z* 257 [M+Na]^+^, C_15_H_22_O_2_. ^1^H-NMR (600 MHz, CDCl_3_) *δ*_H_: 2.67 (1H, dd, *J* = 16.6, 6.1 Hz, H-2a), 2.73 (1H, dd, *J* = 12.0, 6.4 Hz, H-2b), 1.75 (1H, ddt, *J* = 13.7, 6.6, 1.7 Hz, H-3a), 2.04 (1H, ddd, *J* = 13.7, 12.1, 6.8 Hz, H-3b), 4.47 (1H, s, H-5), 7.05 (1H, d, *J* = 7.9 Hz, H-8), 7.10 (1H, d, *J* = 7.9 Hz, H-9), 3.46 (1H, m, H-11), 1.23 (3H, d, *J* = 6.8 Hz, H-12), 1.29 (3H, d, *J* = 6.8 Hz, H-13), 2.20 (3H, s, H-14), 1.41 (3H, s, H-15); ^13^C-NMR (150 MHz, CDCl_3_) *δ*_C_: 135.6 (C-1), 24.8 (C-2), 29.7 (C-3), 72.0 (C-4), 71.3 (C-5), 148.3 (C-6), 134.4 (C-7), 123.9 (C-8), 130.0 (C-9), 134.2 (C-10), 27.6 (C-11), 24.4 (C-12), 25.4 (C-13), 19.7 (C-14), 28.7 (C-15). Supporting information: Figures S37 and S38.

Anomallenodiol (**6**): colorless needle crystal (MeOH), ESI-MS *m*/*z* 261 [M+Na]^+^, C_14_H_22_O_3_. ^1^H-NMR (600 MHz, CD_3_OD) *δ*: 2.22 (1H, m, H-1a), 2.32 (1H, m, H-1b), 1.61 (1H, m, H-2a), 1.70 (1H, ddd, *J* = 13.5, 9.3, 6.1, H-2b), 3.90 (1H, s, H-4), 2.54 (1H, m, H-6), 2.01 (2H, m, H-7), 2.31 (1H, m, H-8a), 2.51 (1H, m, H-8b), 2.22 (1H, m, H-11), 0.94 (3H, d, *J* = 6.9 Hz, H-12), 1.09 (3H, d, *J* = 6.8 Hz, H-13), 1.25 (3H, s, H-15); ^13^C-NMR (150 MHz, CD_3_OD) *δ*: 21.3 (C-1), 31.1 (C-2), 72.0 (C-3), 74.3 (C-4), 160.4 (C-5), 42.7 (C-6), 23.7 (C-7), 36.2 (C-8), 202.4 (C-9), 133.7 (C-10), 30.8 (C-11), 22.1 (C-12), 19.7 (C-13), 25.1 (C-14). Supporting information: Figures S39 and S40.

Oxyphyllenone A (**7**): colorless oil, ESI-MS *m*/*z* 233 [M+Na]^+^, C_12_H_18_O_3_. ^1^H-NMR (400 MHz, CDCl_3_) *δ* 1.44 (1H, m, H-1a), 1.84 (1H, td, *J* = 13.8, 4.1 Hz, H-1b), 1.58 (1H, ddd, *J* = 13.9, 6.9, 3.6 Hz, H-2a), 2.42 (1H, tdd, *J* = 14.1, 4.1, 2.6 Hz, H-2b), 3.70 (1H, t, *J* = 3.0 Hz, H-3), 6.02 (3H, s, H-6), 2.33 (1H, ddd, *J* = 17.6, 3.4, 2.5Hz, H-8a), 2.64 (1H, ddd, *J* = 17.6, 15.2, 5.0 Hz, H-8b), 1.75 (1H, ddd, *J* = 13.2, 5.0, 2.3 Hz, H-9a), 1.93 (1H, m, H-9b), 1.43 (3H, s, H-14), 1.48 (3H, s, H-15); ^13^C-NMR (150 MHz, CDCl_3_) *δ*_C_: 35.3 (C-1), 25.4 (C-2), 76.2 (C-3), 73.9 (C-4), 172.6 (C-5), 126.1 (C-6), 203.4 (C-7), 34.9 (C-8), 41.0 (C-9), 25.5 (C-14), 25.6 (C-15). Supporting information: Figures S41 and S42.

Stachytriol (**8**): cubic crystal (MeOH), ESI-MS *m*/*z* 279 [M+Na]^+^, C_15_H_28_O_3._^1^H NMR (600 MHz, CDCl_3_) *δ*: 1.33 (1H, m, H-2a), 2.41 (1H, m, H-2b), 1.39 (1H, m, H-3a), 1.88 (1H, m, H-3b), 2.55 (1H, m, H-4), 1.79 (1H, m, H-5), 1.48 (1H, m, H-6a), 1.57 (1H, m, H-6b), 1.79 (1H, m, H-7), 1.63 (1H, m, H-8a), 2.02 (1H, m, H-8b), 1.63 (1H, m, H-9a), 2.02 (1H, m, H-9b), 1.24 (3H, s, H-12), 1.14 (3H, s, H-13), 1.05 (3H, s, H-14), 0.89 (3H, d, *J* = 6.9 Hz, H-15); ^13^C NMR (150 MHz, CDCl_3_) *δ*: 90.7 (C-1), 32.3 (C-2), 29.6 (C-3), 35.4 (C-4), 47.9 (C-5), 24.5 (C-6), 36.5 (C-7), 19.3 (C-8), 26.2 (C-9), 75.8 (C-10), 73.7 (C-11), 29.4 (C-12), 29.2 (C-13), 26.8 (C-14), 15.1 (C-15). Supporting information: Figures S43 and S44.

### 3.4. X-Ray Crystallographic Analysis of Compounds **1**, **2** and **4**

Crystals of compounds **1**, **2** and **4** were obtained by the slow evaporation of a CHCl_3_–MeOH (1:4) solution at room temperature over four days. These crystals were analyzed using a XtaLAB AFC12 (RINC) diffractometer (Rigaku Corporation, Tokyo, Japan) equipped with Cu Kα radiation during crystal data collection. Using Olex2 version 1.5 [22], the structure was solved with the SHELXT [23] structure solution program using intrinsic phasing and refined with the SHELXL [24] refinement package using least-squares minimization. The crystallographic data of **1**, **2** and **4** have been deposited in the Cambridge Crystallographic Data Center (http://www.ccdc.cam.ac.uk, accessed on 15 September 2024).

Crystal data for Migaone A (**1**): C_15_H_24_O_3_ *M* = 252.34, orthorhombic, space group P2_1_2_1_2_1_ (no. 19), *a* = 18.0068 (3) Å, *b* = 10.4598 (2) Å, *c* = 7.3920 (2) Å, *α* = 90°, *β* = 90°, *γ* = 90°, *V* = 1392.26 (5) Å^3^, *Z* = 4, *T* = 150.3 (4) K, *μ* (Cu Kα) = 0.654 mm^−1^, 12,977 reflections measured (9.78° ≤ 2Θ ≤ 147.7°), 2733 unique (*R*_int_ = 0.0416, *R*_sigma_ = 0.0315), which were used in all calculations. The final *R*_1_ was 0.0328 (I > 2σ(I)) and *wR*_2_ was 0.0824 (all data). Flack parameter = −0.02(9). CCDC number: 2377460.

Crystal data for Migaone B (**2**): C_15_H_20_O_2_, *M* = 232.31, monoclinic, space group P2_1_ (no. 4), *a* = 11.2012 (10) Å, *b* = 10.0306 (10) Å, *c* = 12.5157 (10) Å, *α* = 90°, *β* = 112.1760°, *γ* = 90°, *V* = 1302.18 (2) Å^3^, *Z* = 4, *T* = 99.98 (11) K, *μ* (Cu Kα) = 0.605 mm^−1^, 24,883 reflections measured (7.628° ≤ 2Θ ≤ 148.854°), 5208 unique (*R*_int_ = 0.0303, *R*_sigma_ = 0.0187) which were used in all calculations. The final *R*_1_ was 0.0309 (I > 2σ(I)) and *wR*_2_ was 0.0818 (all data). Flack parameter = 0.00 (5). CCDC number: 2377461.

Crystal data for Migaone D (**4**): C_15_H_24_O_2_, *M* = 236.34, monoclinic, space group P2_1_ (no. 4), *a* = 6.6487 (11) Å, *b* = 13.674 (**2**) Å, *c* = 14.980 (4) Å, *α* = 90°, *β* = 95.739°, *γ* = 90°, *V* = 1355.1 (5) Å^3^, *Z* = 2, *T* = 99.98 (16) K, *μ* (Cu Kα) = 0.582 mm^−1^, 6393 reflections measured (5.93° ≤ 2Θ ≤ 149.358°), 3435 unique (*R*_int_ = 0.0647, *R*_sigma_= 0.0898), which were used in all calculations. The final *R*_1_ was 0.0722 (I > 2σ(I)) and *wR*_2_ was 0.2144 (all data). Flack parameter = 0.00 (5). CCDC number: 2377462.

### 3.5. Theoretical ECD Calculation

The absolute configuration of compound **3** were determined using time-dependent density functional theory (TDDFT) calculations, which were performed with the Gaussian 16 program package. The stable conformers, devoid of imaginary frequencies, were subjected to ECD calculation using the TDDFT method at the B3LYP-SCRF (PCM)/6-31+G (d) levels with the CPCM model in methanol solvent. The final calculated ECD spectrum was obtained by Boltzmann averaging, and the calculated ECD of each conformer using SpecDis 1.64. The absolute configurations of compound **3** were determined by comparing the calculated ECD with the experimental ECD data [25].

### 3.6. Cell Viability Assay

Differentiated PC12 cells (purchased from Institute of Cell Biology, Chinese Academy of Sciences, Shanghai, China) were cultured in Dulbecco’s Modified Eagle Medium (DMEM) supplemented with 5% fetal bovine serum and 1% penicillin–streptomycin. The cells were incubated at 37 °C in a humidified environment containing 5% CO_2_ based on a previously reported method [21]. All tested compounds were dissolved in dimethyl sulfoxide (DMSO). Compounds **1**–**4** were evaluated for their neuroprotective effects against NMDA-induced neurotoxicity utilizing the MTT assay [26]. PC12 cells were plated at a density of 4000 cells/well in a 96-well plate. After 72 h of incubation, the cells were pretreated with MK-801 (positive control) and compounds **1**–**4** at 30 µM for 24 h, which was followed by treatment with NMDA (2 mM) for 6 h. Subsequently, 3-(4,5-dimethylthiazol-2-yl)-2,5-diphenyltetrazolium bromide (MTT) was added to the medium at a final concentration of 0.5 mg/mL. After 4 h incubation, the formazan crystals were dissolved in 100 µL DMSO per well, and the absorbance was measured at a wavelength of 490 nm using a Microplate Reader (Thermo Scientific Varioskan LUX Multimode Reader, Thermo Scientific Co., Ltd., Waltham, MA, USA). Data were processed using SPSS 27.0, and graphs were generated using GraphPad Prism 8.

### 3.7. Molecular Docking Study

The Autodock 4 version software package was used for the docking study between the ligand and protein. The NMDAR protein (PDB code: 4NF4) was downloaded from Protein Data Bank (https://www.rcsb.org/, accessed on 15 September 2024). For active site docking, a grid box of size 30 × 30 × 30 Å was defined with the center coordinates set at X = −13.559, Y = 7.812, Z = −37.745. The pose with the best score was selected for further analysis, which was performed visually using PyMoL version 2.4. Additionally, two-dimensional images were generated using the LIGPLOT^+^ Molecular Graphics System 2.2.5.

### 3.8. Statistical Analysis

Data are expressed as the mean ± standard deviation (SD). Multiple groups were compared using one-way analysis of variance (ANOVA), which was followed by the post hoc least significant difference (LSD) test. A *p*-value of less than 0.05 was considered statistically significant.

## 4. Conclusions

In conclusion, four new sesquiterpenes, Migaones A–D (**1**–**4**), along with four known compounds (**5**–**8**), were isolated from the branches and leaves of *C. migao*. Except for compound **2**, all other isolated constituents possess vicinal diol structural units. Bioactivity assessments revealed that compounds **1**–**2** exhibited protective effects against NMDA-induced neurotoxicity in PC12 cells at a concentration of 30 μM. Furthermore, molecular docking analyses demonstrated that compounds **1**–**2** effectively interacted with the glycine binding pocket through hydrogen bonds and hydrophobic interactions with key amino acid residues. These molecular docking results were consistent with the observed biological activity.

## Figures and Tables

**Table 1:** NMR data for Migaone A−D (**1**−**4**).

No.	Migaone A (1) ^a^		Migaone B (2) ^a^		Migaone C (3) ^b^		Migaone D (4) ^b^	
	*δ*_C_	*δ*_H_ (*J* in Hz)	*δ*_C_	*δ*_H_ (*J* in Hz)	*δ*_C_	*δ*_H_ (*J* in Hz)	*δ*_C_	*δ*_H_ (*J* in Hz)
1	33.0	1.85, m	35.9	2.02, td (14.0, 5.3)	51.5	2.15, m	132.4	
		1.20, m		1.82, ddd (13.3, 5.3, 2.3)				
2	17.4	1.85, m	33.7	2.62, ddd(17.8, 14.5, 5.2)	26.2	1.78, m	25.6	2.30, m
		1.50, m		2.55, ddd (17.9, 5.2, 2.3)		1.70, m		2.24, m
3	36.3	1.97, m	198.6		35.1	1.70, m	31.9	1.86, m
		1.50, m				1.26, m		1.52, m
4	74.4		133.3		37.5	2.23, m	71.4	
5	73.8		152.1		62.0	1.93, dd (10.7, 7.8)	73.2	3.78, s
6	139.7	6.87, s	133.6	7.16, s	207.3		139.1	
7	148.5		148.8		147.6		43.1	2.43, m
8	199.8		197.9		141.5	6.16, dd (4.6, 1.2)	24.0	1.70, m
								1.52, m
9	51.2	2.91, m	53.0	2.45, m	76.0	4.17, d (4.6)	31.5	2.43, m
		1.98, m						2.24, m
10	41.3		37.5		76.0		146.0	
11	26.4	2.90, m	27.2	3.02, p (6.9)	30.1	2.82, m	29.7	2.17, m
12	22.1	1.05, d (6.9)	22.0	1.11, d (6.9)	22.1	1.04, d, (6.9)	18.2	0.76, d (6.9)
13	21.5	1.07, d (6.9)	21.8	1.14, d (6.9)	22.6	0.97, d, (6.9)	21.9	0.97, d (6.9)
14	23.6	1.23, s	23.9	1.25, s	19.9	1.24, s	108.5	4.92, s
								4.74, s
15	26.7	1.37, s	11.2	1.97, s	21.3	1.09, d (6.7)	24.7	1.15, s

^a^ Spectra were measured in CDCl_3_ (^1^H, 600 MHz and ^13^C, 150 MHz); ^b^ Spectra were measured in CD_3_OD (^1^H, 600 MHz and ^13^C, 150 MHz).

## Data Availability

The data presented in this study are available on request from the corresponding author.

## Supplementary Materials

The supporting information can be downloaded at: https://www.mdpi.com/article/10.3390/ijms252312693/s1.
