# Supplementary material for: Vicinal Diol Sesquiterpenes from Cinnamomum migao with Neuroprotective Effects in PC12 Cells
Source: Int J Mol Sci. 2024 Nov 26;25(23):12693. doi: 10.3390/ijms252312693 (PMC11641330; doi:10.3390/ijms252312693)

Supporting Information

Vicinal diol sesquiterpenes from *Cinnamomum migao* with neuroprotective effects in PC12 cells

Lang Zhou [1,2], Fa-Ju Chen [1,2], Li-Shou Yang [1,2], Mei Peng [1,2], Xiong Pan [1,2], Hua-Yong Lou [1,2], Juan Yang [1,2], Xiao-Sheng Yang [1,2*], Qi-Ji Li [1,2*]

*Corresponding author

Correspondence: yangxiaosheng@gmc.edu.cn (X.Y.); liqiji@gmc.edu.cn (Q.L.).

[1] State Key Laboratory of Functions and Applications of Medicinal Plants, Guizhou Medical University, Gaohai road, Guiyang, 550014, Guizhou, China.

[2] Natural Products Research Center of Guizhou Province, Gaohai road, Guiyang, 550014, Guizhou, China.

Table of Contents

Figure S1. ^{1}H-NMR (600 MHz, $CDCl_3$) spectrum of **1.** 4

Figure S2. ^{13}C-NMR (150 MHz, $CDCl_3$) spectrum of **1**. 4

Figure S3. HSQC spectrum of **1**. 5

Figure S4. HMBC spectrum of **1**. 5

Figure S5. ^{1}H-^{1}H COSY spectrum of **1**. 6

Figure S6. NOESY spectrum of **1**....6

Figure S7. HR-ESI-MS spectrum of **1**. 7

Figure S8. UV spectrum of **1**. 7

Figure S9. IR spectrum of **1**. 8

Figure S10. ^{1}H-NMR (600 MHz, $CDCl_3$) spectrum of **2**. 8

Figure S11. ^{13}C-NMR (150 MHz, $CDCl_3$) spectrum of **2**....9

Figure S12. HSQC spectrum of **2**. 9

Figure S13. HMBC spectrum of **2**. 10

Figure S14. ^{1}H-^{1}H COSY spectrum of **2**. 10

Figure S15. NOESY spectrum of **2**....11

Figure S16. HR-ESI-MS spectrum of **2**. 11

Figure S17. UV spectrum of **2**. 12

Figure S18. IR spectrum of **2**. 12

Figure S19. ^{1}H-NMR (600 MHz, CD_3OD) spectrum of **3**. 13

Figure S20. ^{13}C-NMR (150 MHz, CD_3OD) spectrum of **3**....13

Figure S21. HSQC spectrum of **3**. 14

Figure S22. HMBC spectrum of **3**. 14

Figure S23. ^{1}H-^{1}H COSY spectrum of **3**. 15

Figure S24. NOESY spectrum of **3**....15

Figure S25. HR-ESI-MS spectrum of **3**. 16

Figure S26. UV spectrum of **3**. 16

Figure S27. IR spectrum of **3**. 17

Figure S28. ^{1}H-NMR (600 MHz, CD_3OD) spectrum of **4**. 17

Figure S29. ^{13}C-NMR (600 MHz, CD_3OD) spectrum of **4**....18

Figure S30. HSQC spectrum of **4**. 18
Figure S31. HMBC spectrum of **4**. 19
Figure S32. ^{1}H-^{1}H COSY spectrum of **4**. 19
Figure S33. NOESY spectrum of **4**. 20
Figure S34. HR-ESI-MS spectrum of **4**. 20
Figure S35. UV spectrum of **4**. 21
Figure S36. IR spectrum of **4**. 21
Figure S37. ^{1}H-NMR (600 MHz, CD_3OD) spectrum of **5**. 22
Figure S38. ^{13}C-NMR (150 MHz, CD_3OD) spectrum of **5**. 22
Figure S39. ^{1}H-NMR (600 MHz, CD_3OD) spectrum of **6**. 23
Figure S40. ^{13}C-NMR (150 MHz, CD_3OD) spectrum of **6**. 23
Figure S41. ^{1}H-NMR (600 MHz, CD_3OD) spectrum of **7**. 24
Figure S42. ^{13}C-NMR (150 MHz, CD_3OD) spectrum of **7**. 24
Figure S43. ^{1}H-NMR (600 MHz, $CDCl_3$) spectrum of **8**. 25
Figure S44. ^{13}C-NMR (150 MHz, $CDCl_3$) spectrum of **8**. 25

Figure S1. ^{1}H-NMR (600 MHz, $CDCl_3$) spectrum of **1**.

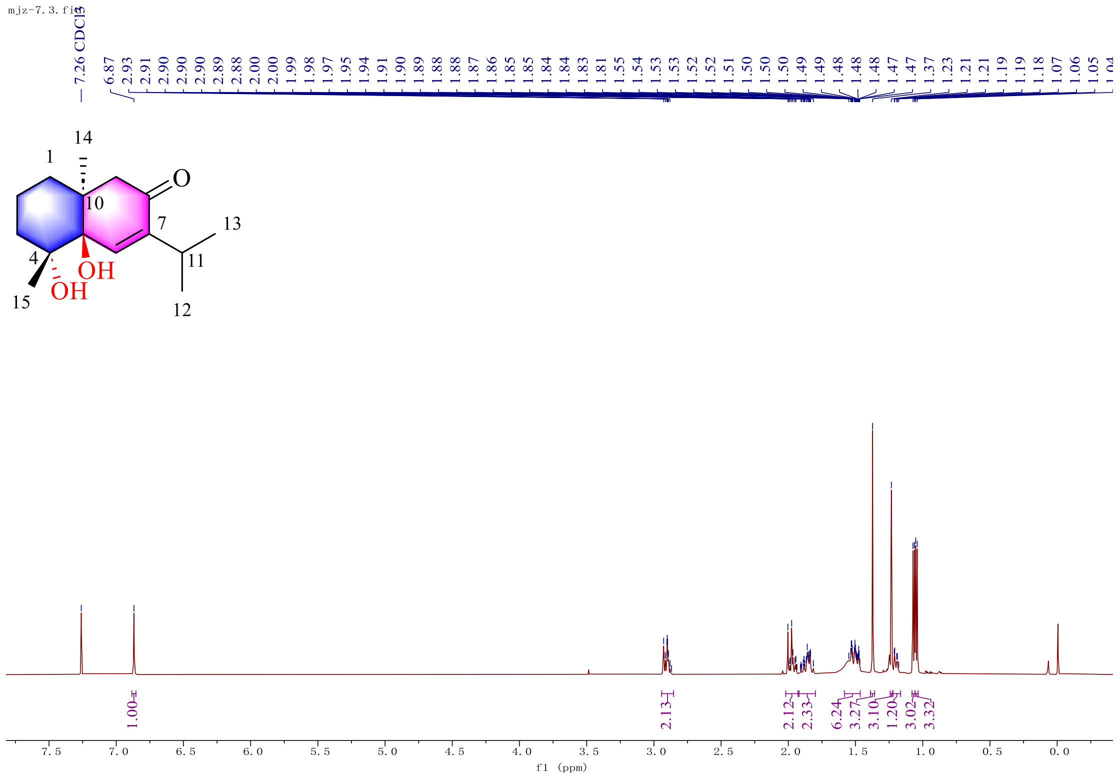

Figure S2. ^{13}C-NMR (150 MHz, $CDCl_3$) spectrum of **1**.

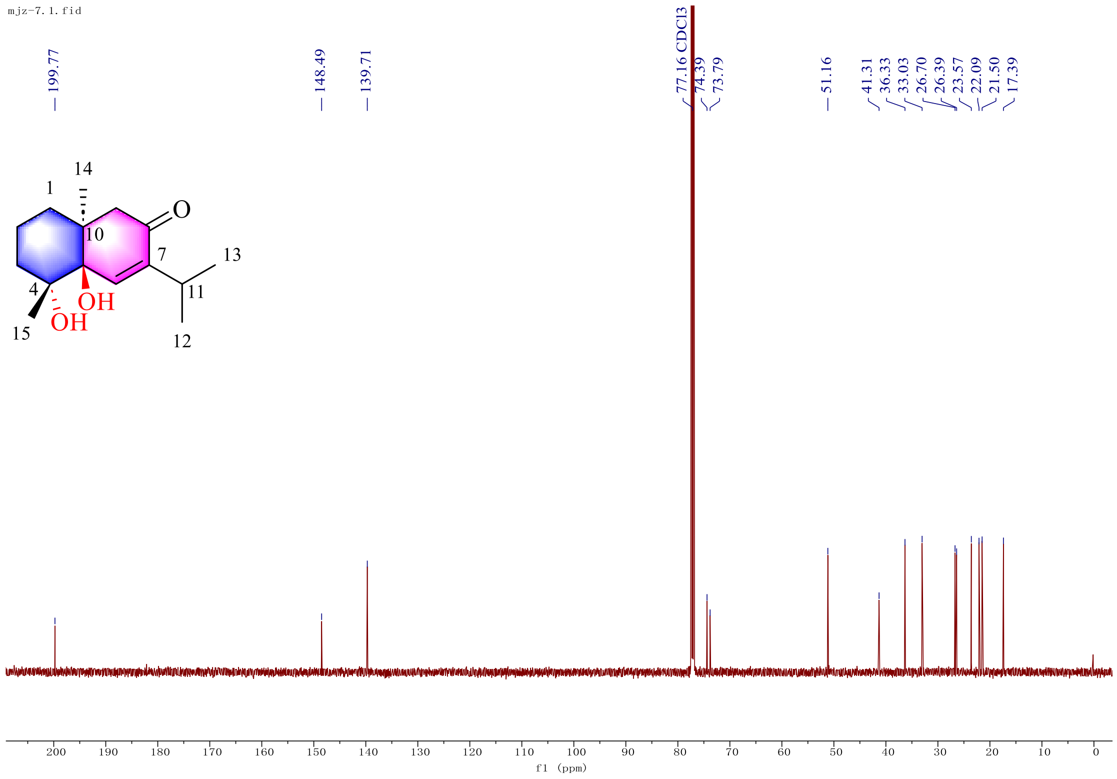

Figure S3. HSQC spectrum of **1**.

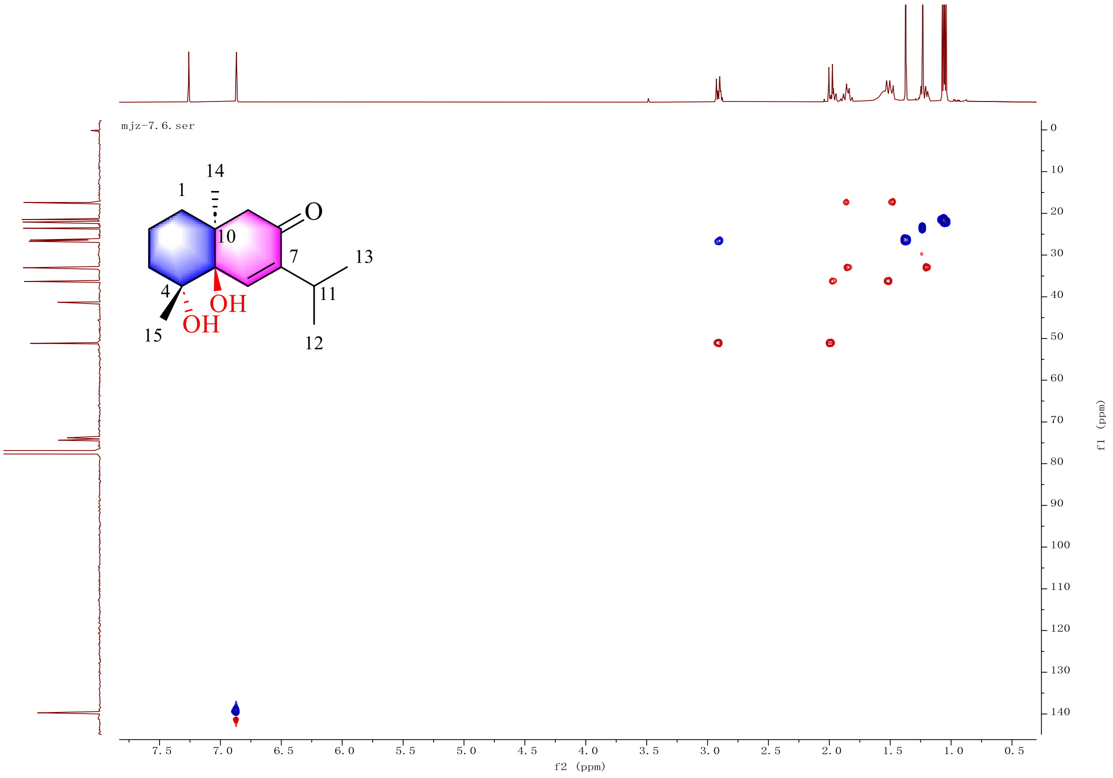

Figure S4. HMBC spectrum of **1**.

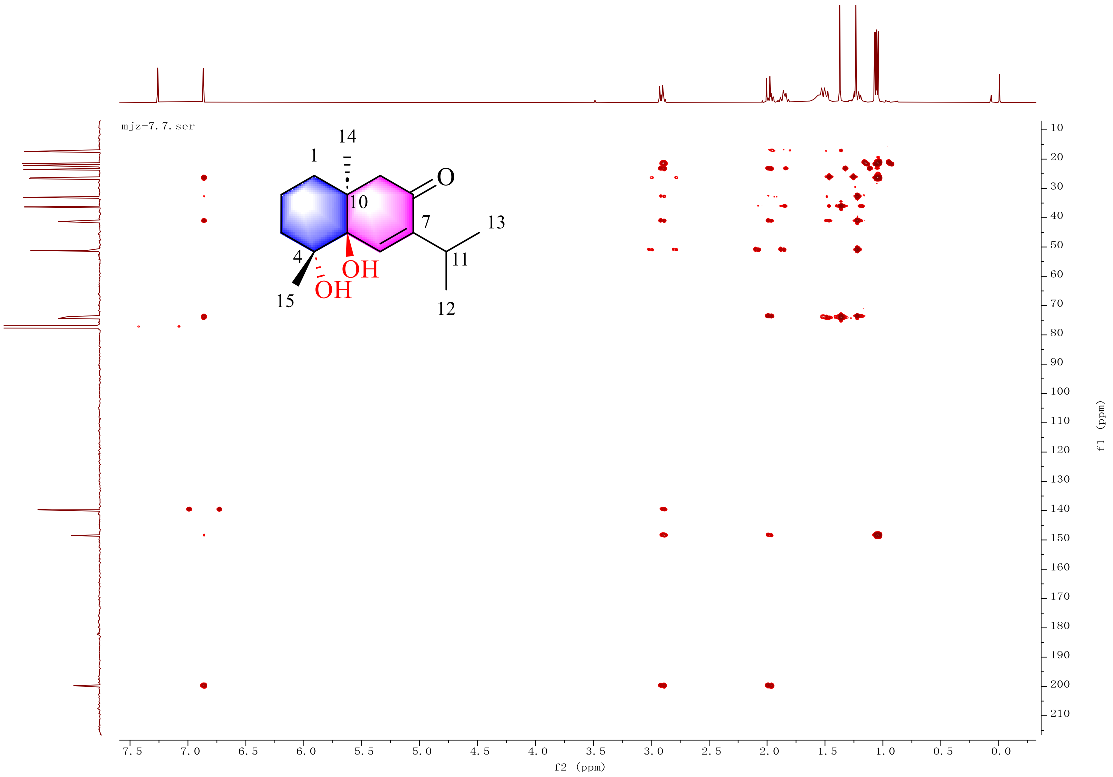

Figure S5. ^{1}H-^{1}H COSY spectrum of **1**.

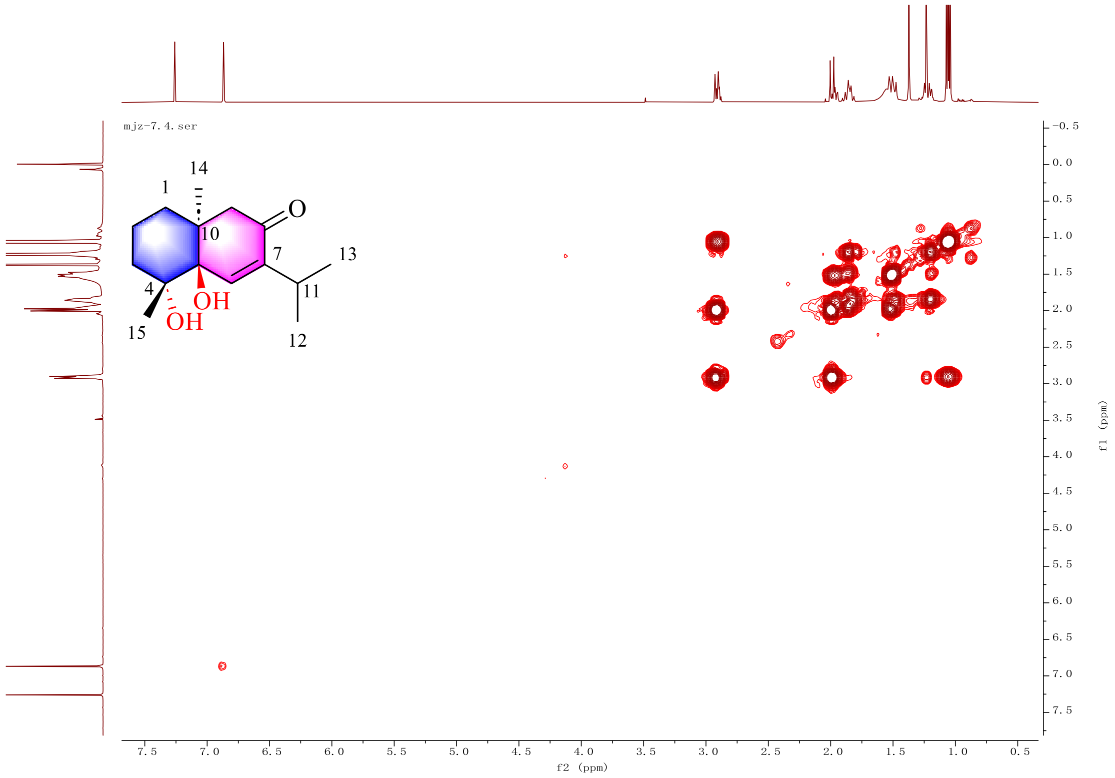

Figure S6. NOESY spectrum of **1**.

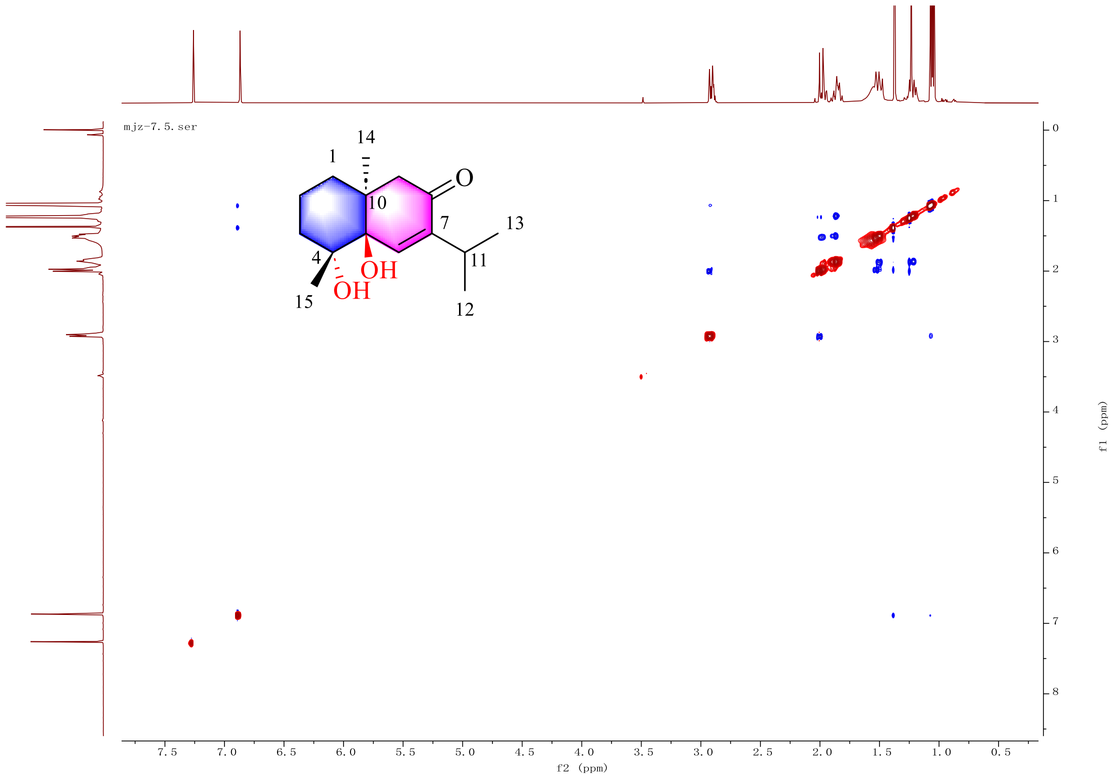

Figure S7. HR-ESI-MS spectrum of **1**.

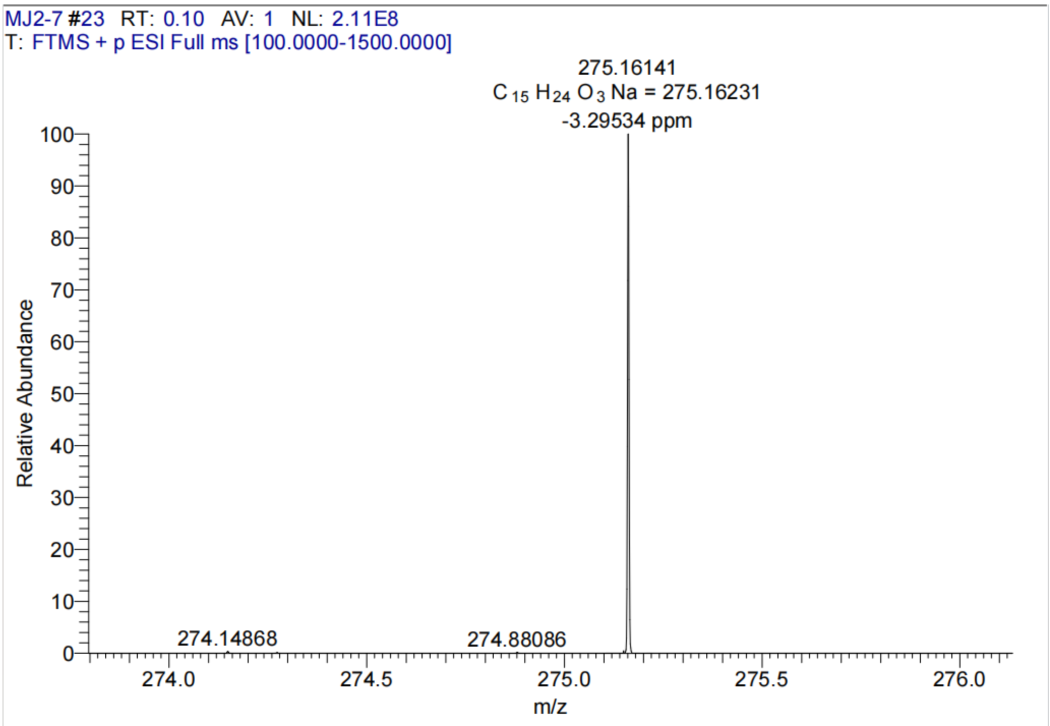

Figure S8. UV spectrum of **1**.

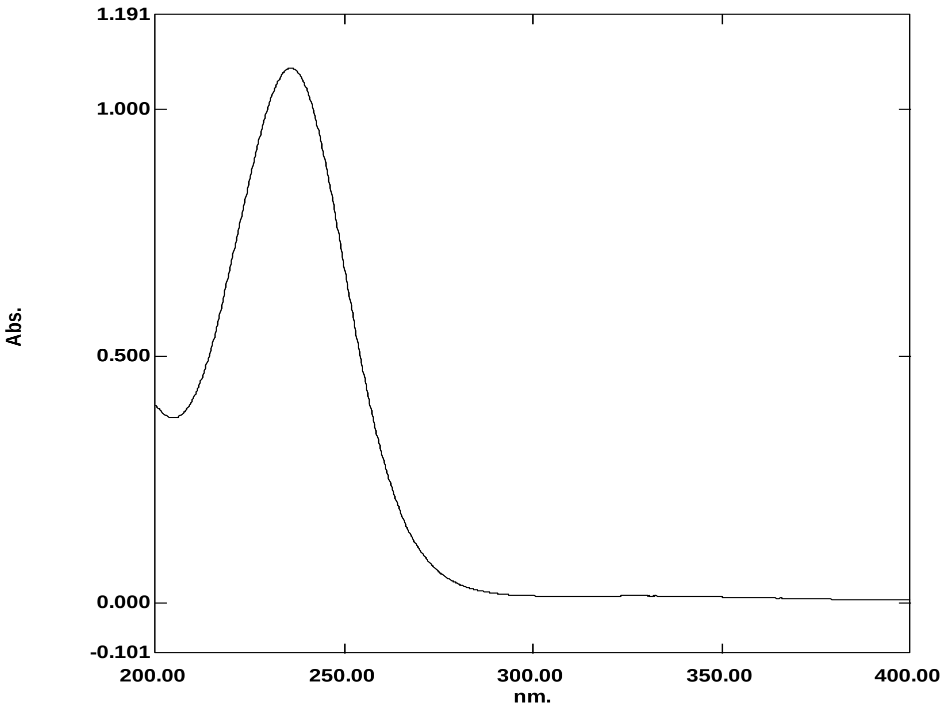

No.	P/V	Wavelength (nm)	Abs.
1		235.80	1.084

Figure S9. IR spectrum of **1**.

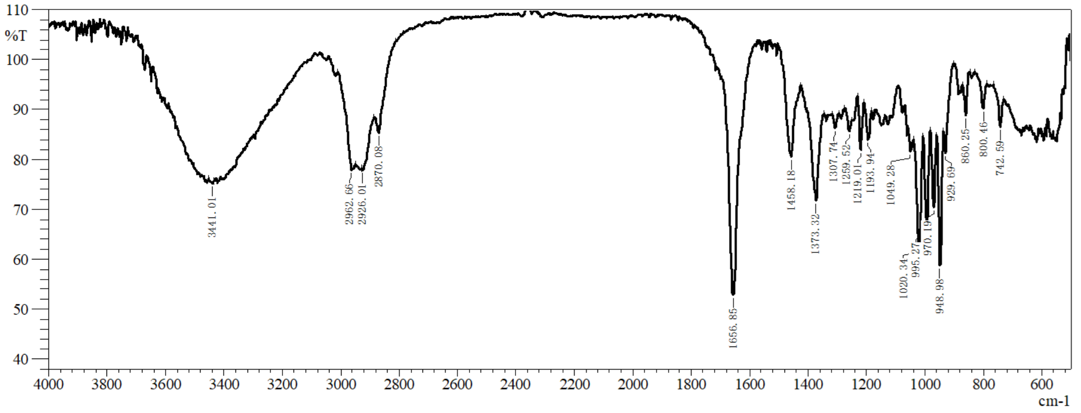

Figure S10. ^{1}H-NMR (600 MHz, $CDCl_3$) spectrum of **2**.

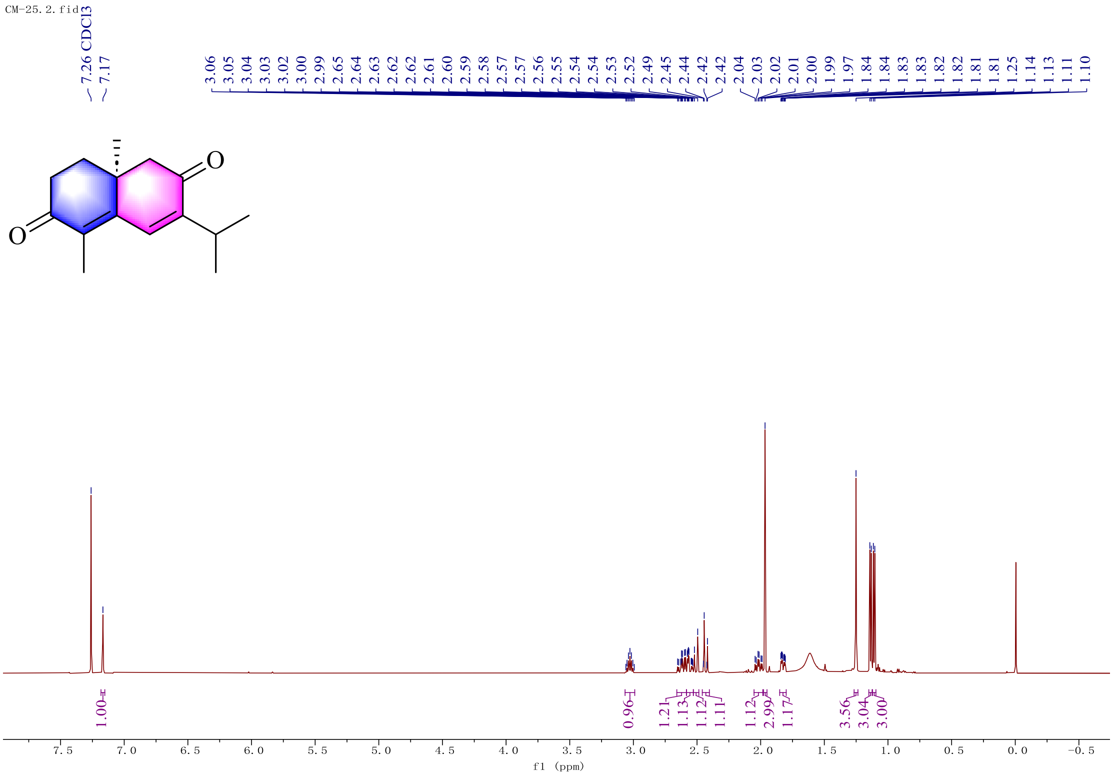

Figure S11. ^{13}C-NMR (150 MHz, $CDCl_3$) spectrum of **2**.

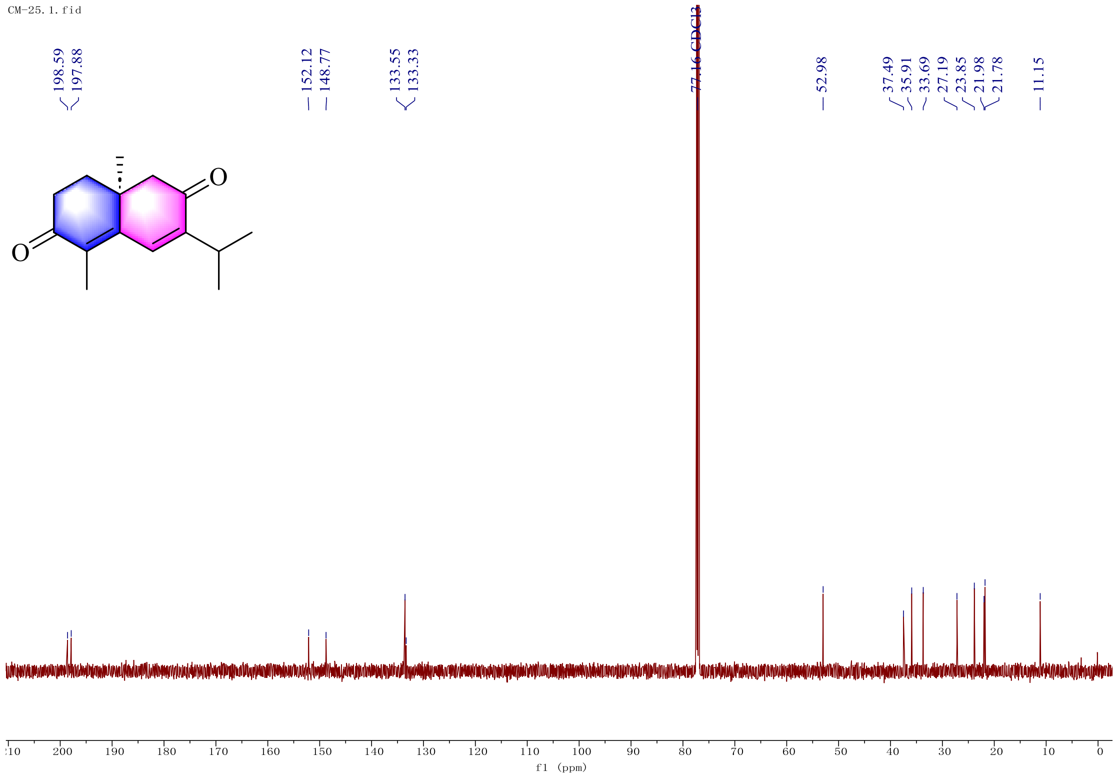

Figure S12. HSQC spectrum of **2**.

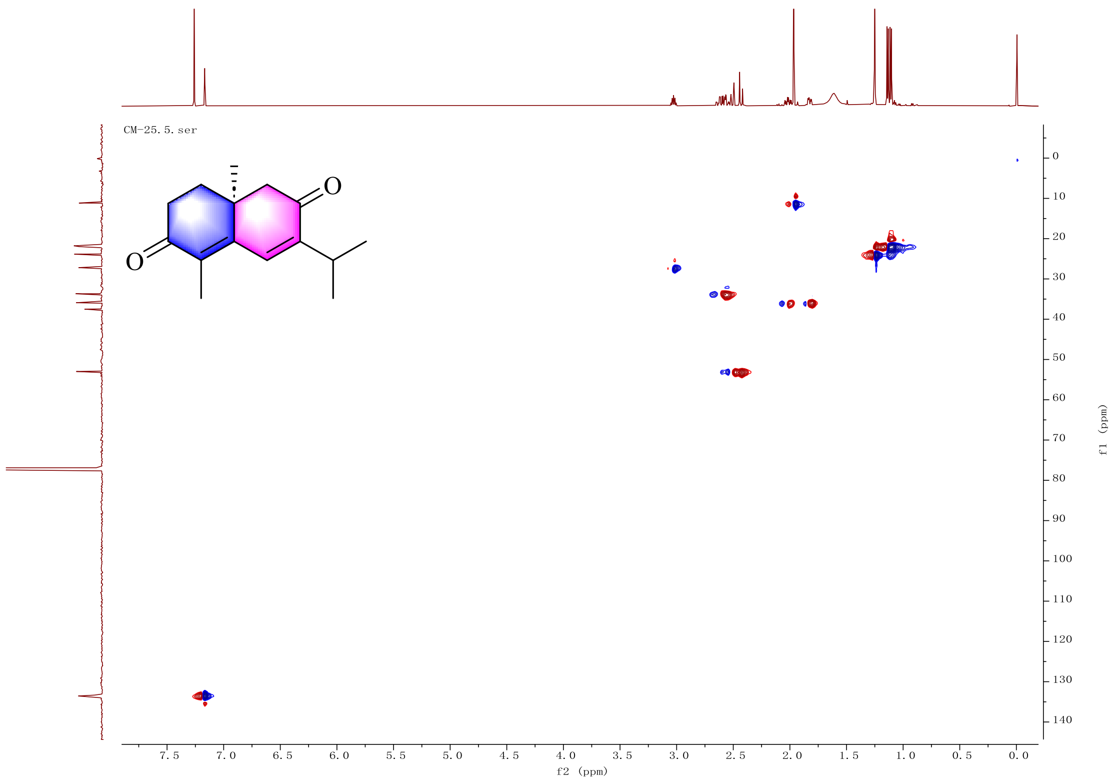

Figure S13. HMBC spectrum of **2**.

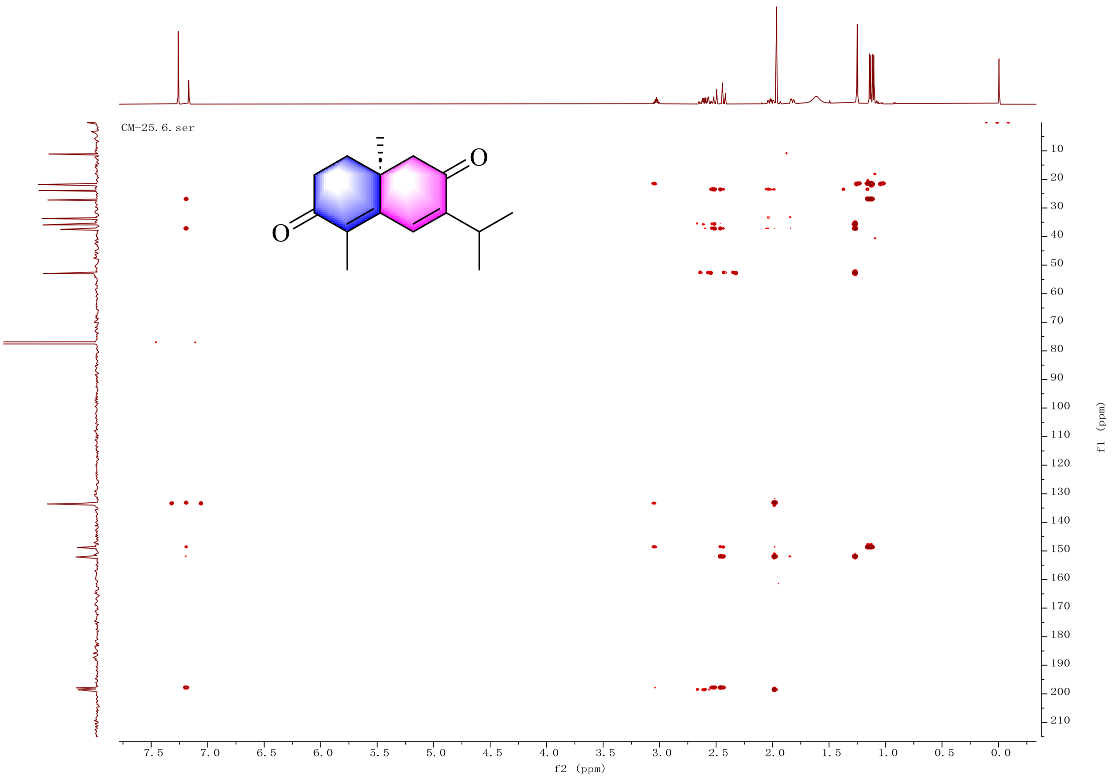

Figure S14. ^{1}H-^{1}H COSY spectrum of **2**.

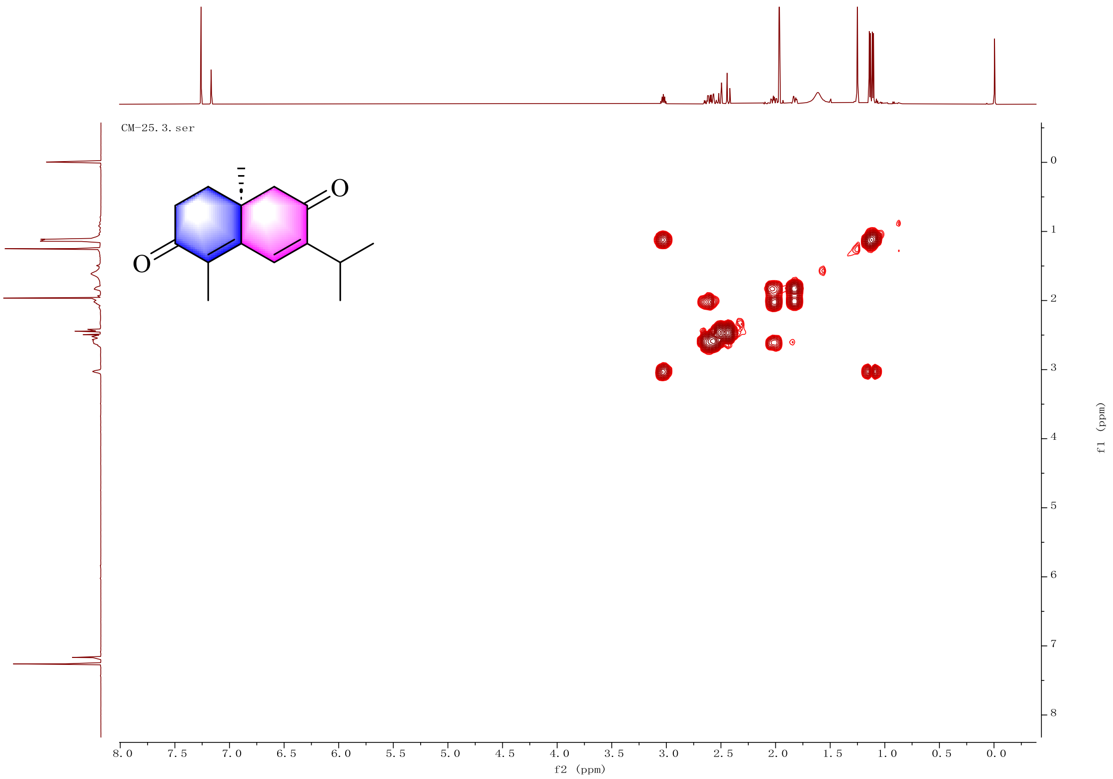

Figure S15. NOESY spectrum of **2**.

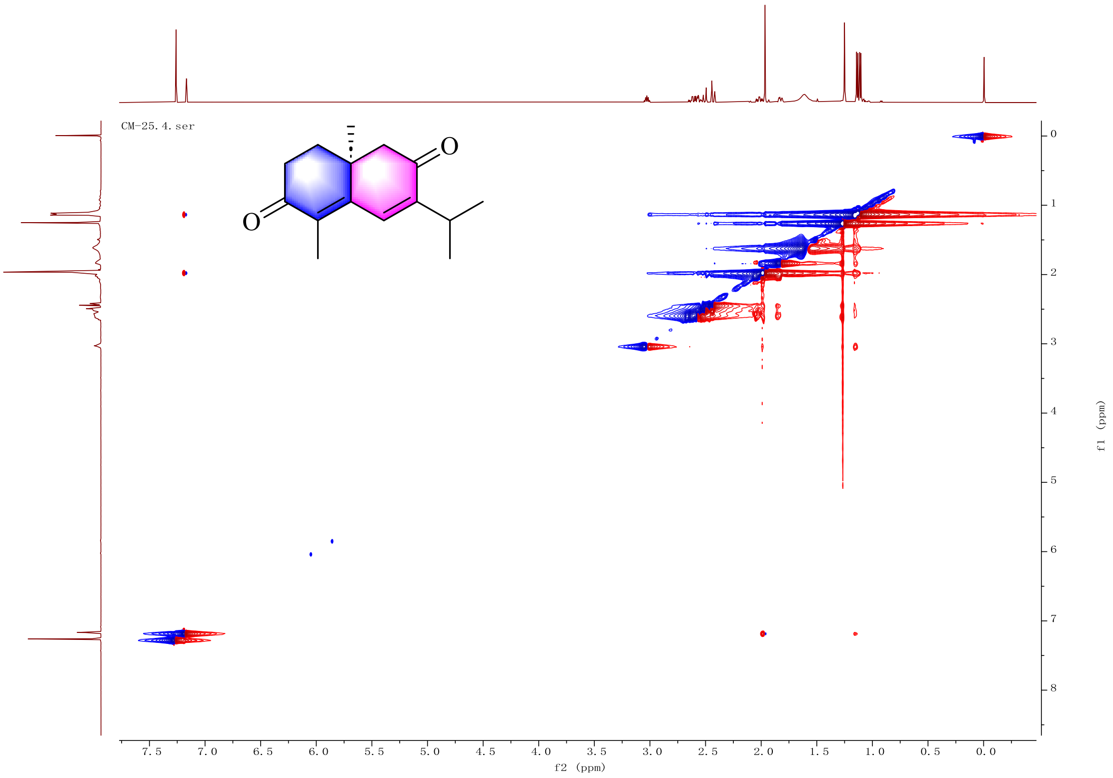

Figure S16. HR-ESI-MS spectrum of **2**.

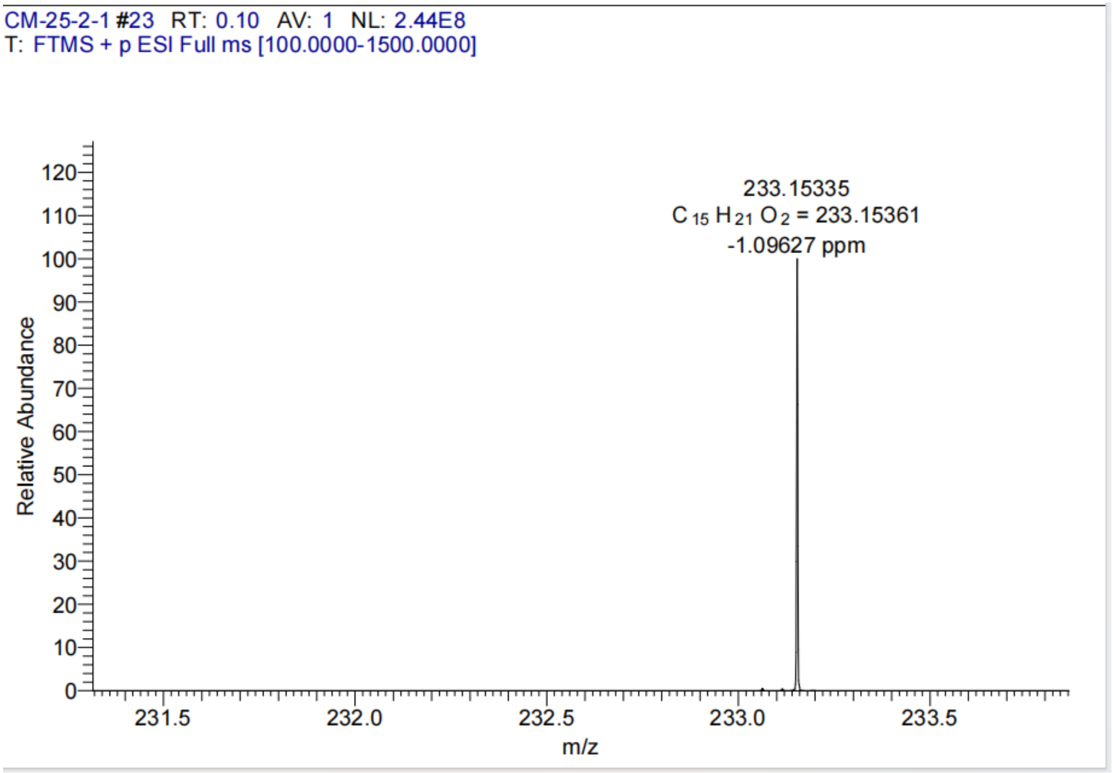

Figure S17. UV spectrum of **2**.

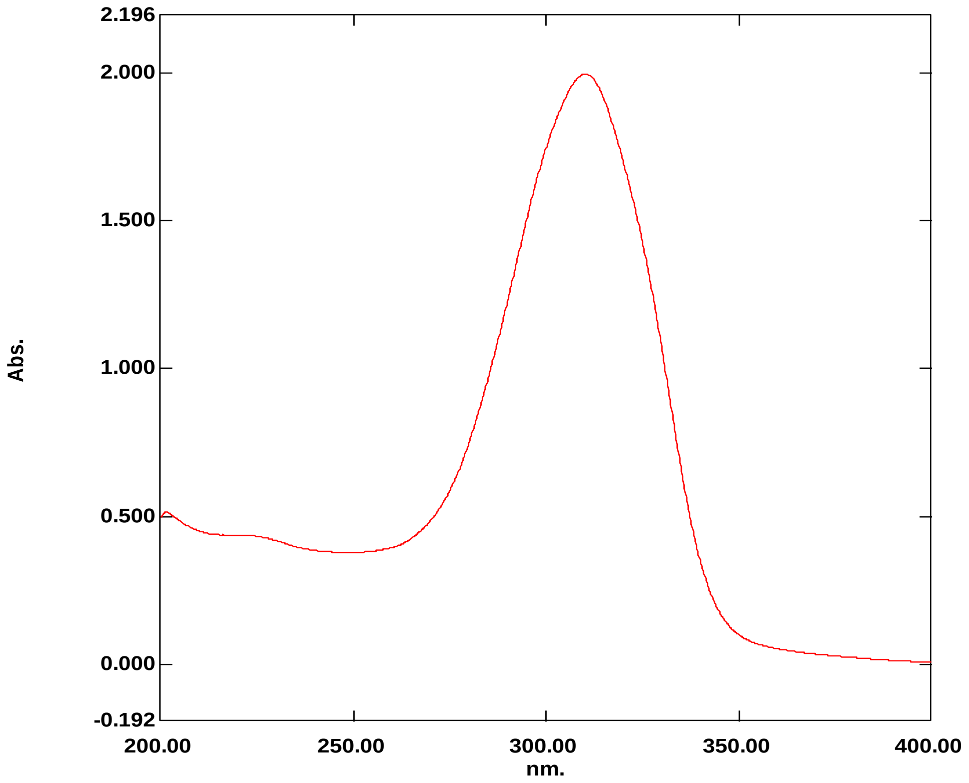

No.	P/V	Wavelength (nm)	Abs.
1		310.00	1.997

Figure S18. IR spectrum of **2**.

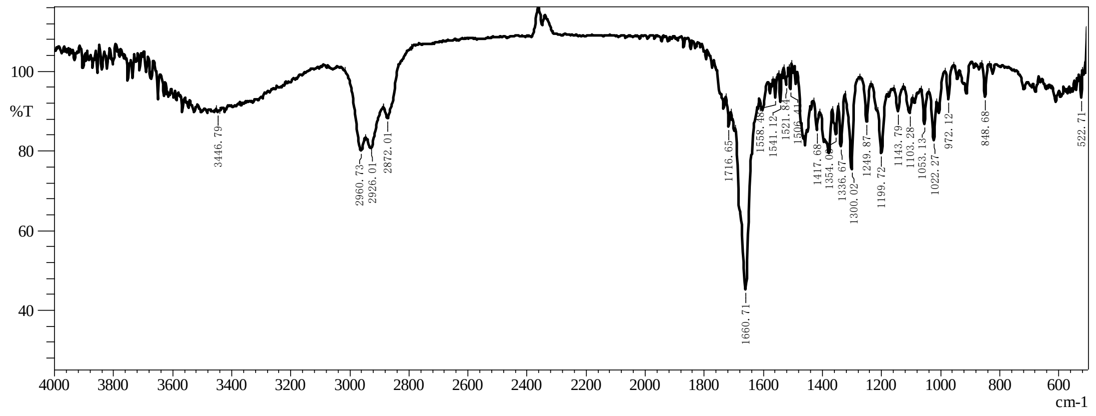

Figure S19. ^{1}H-NMR (600 MHz, CD_3OD) spectrum of **3**.

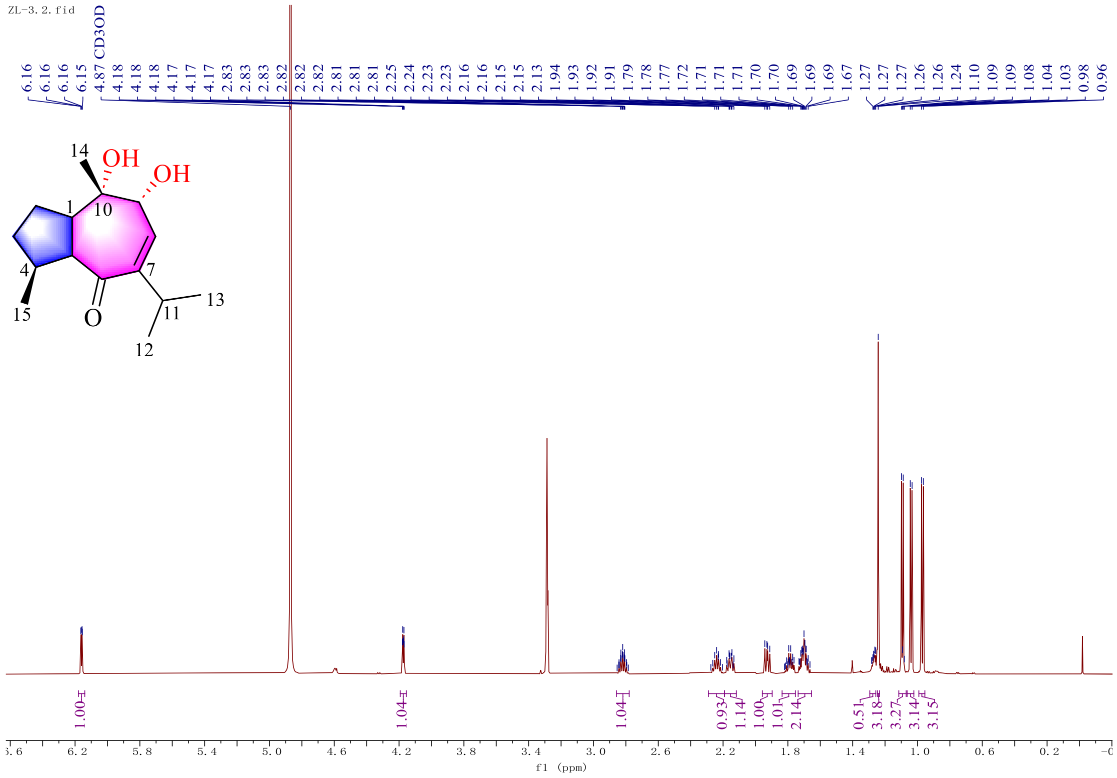

Figure S20. ^{13}C-NMR (150 MHz, CD_3OD) spectrum of **3**.

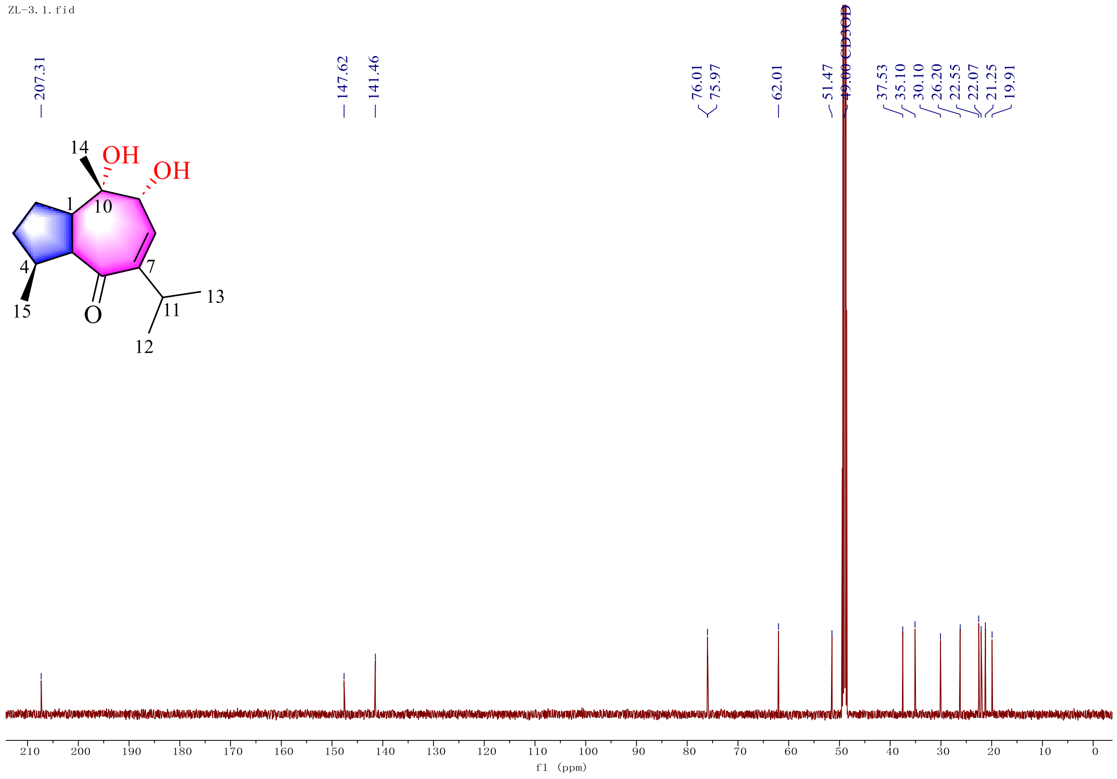

Figure S21. HSQC spectrum of **3**.

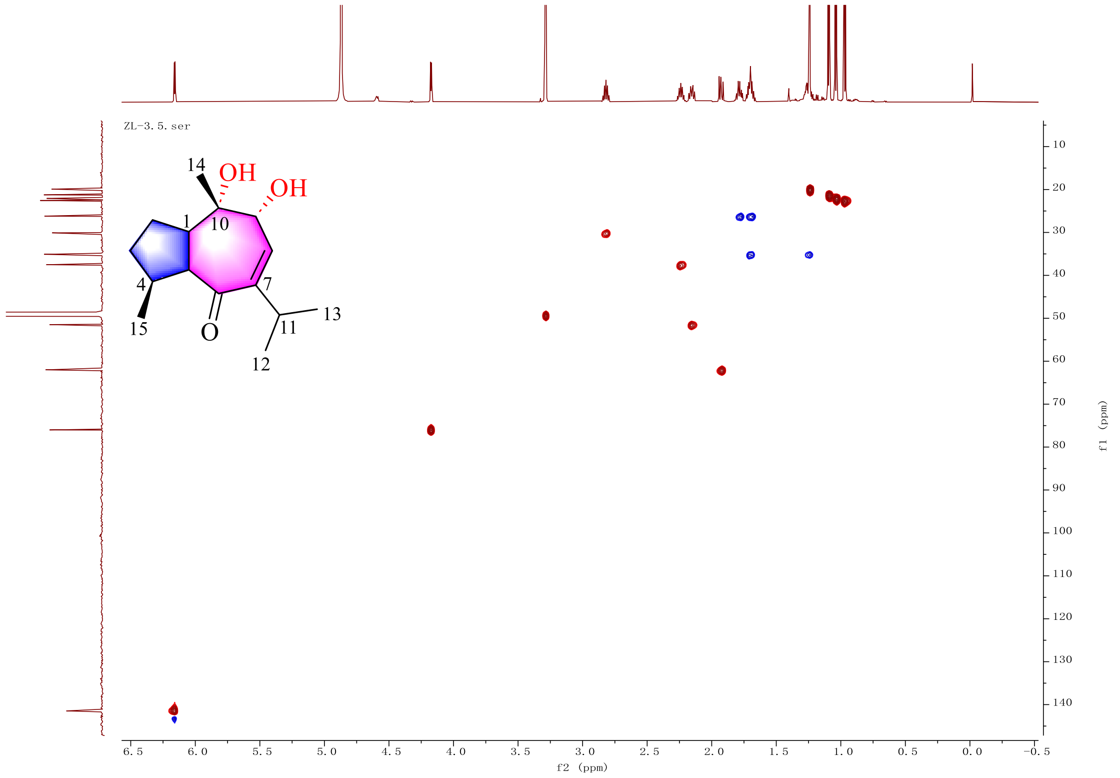

Figure S22. HMBC spectrum of **3**.

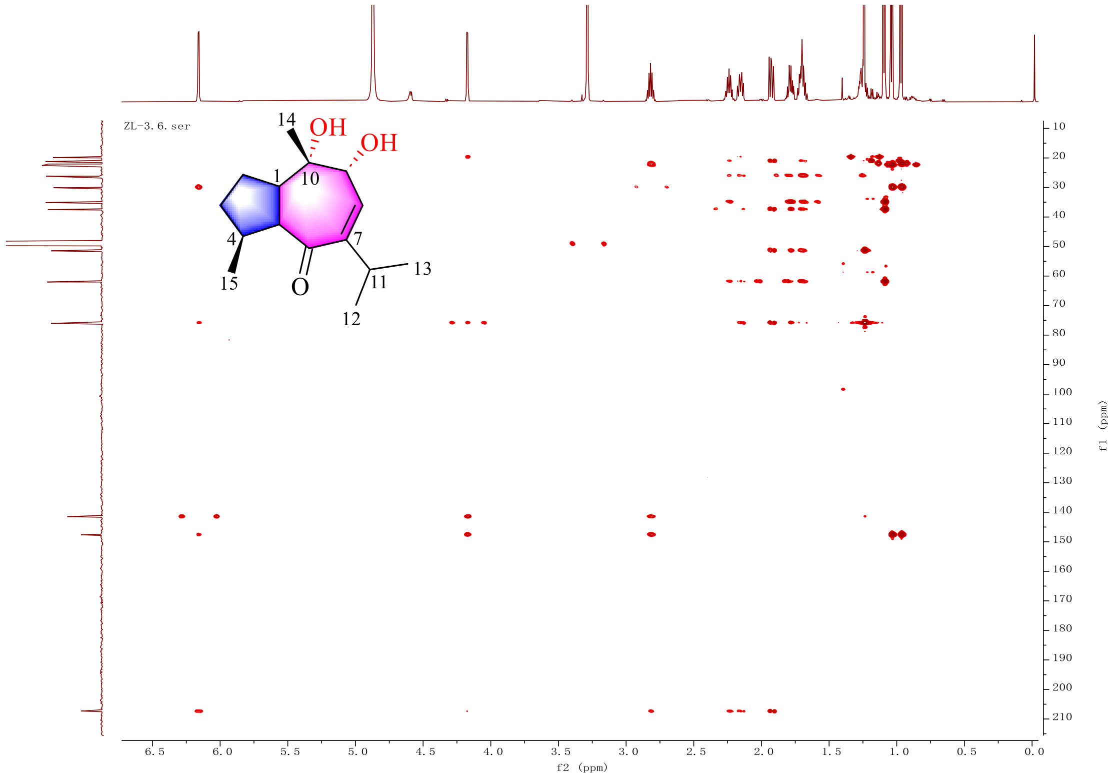

Figure S23. ^{1}H-^{1}H COSY spectrum of **3**.

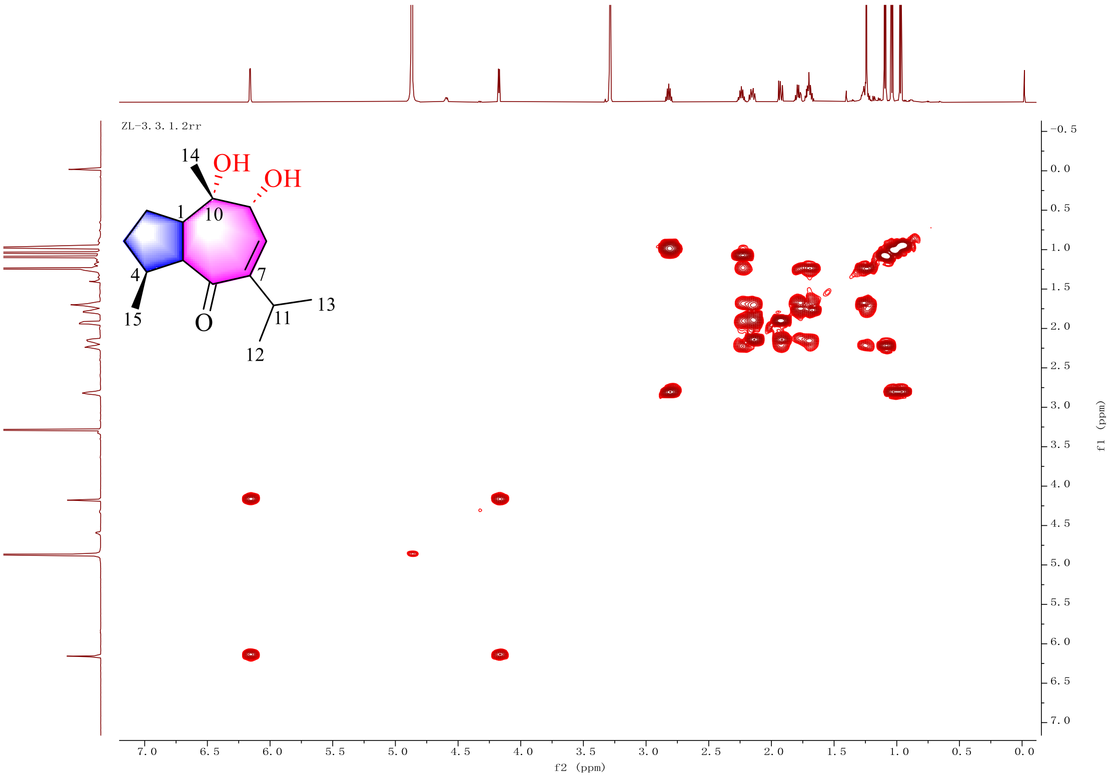

Figure S24. NOESY spectrum of **3**.

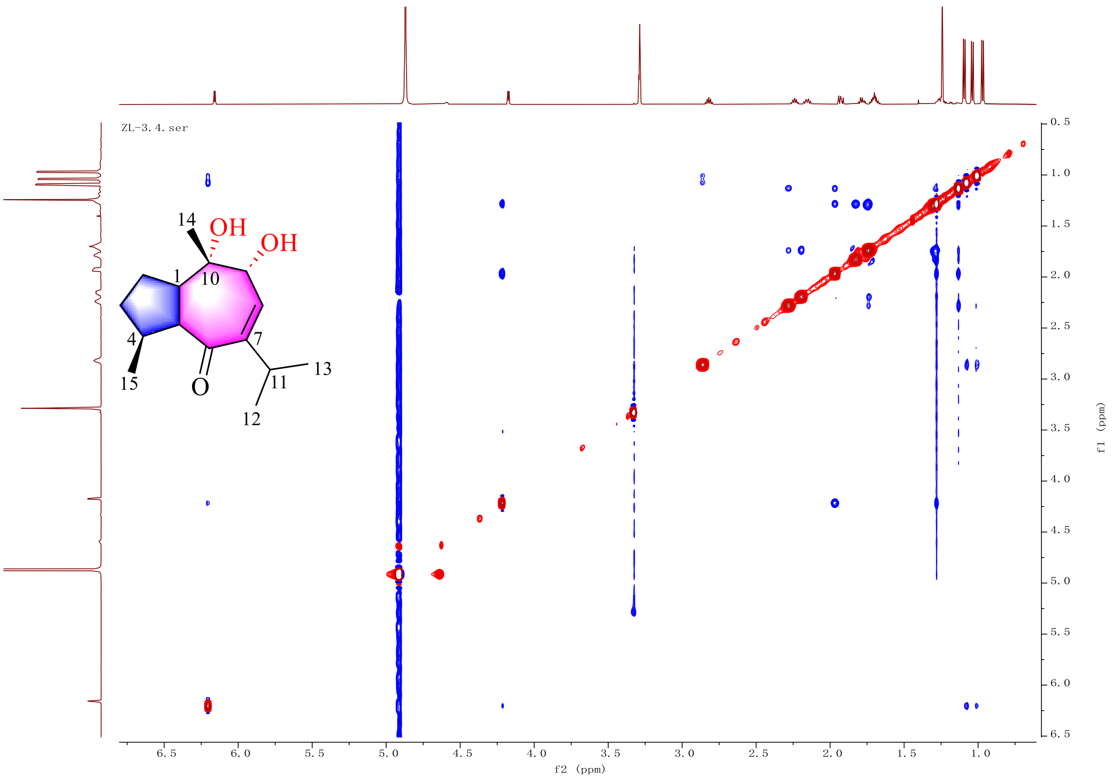

Figure S25. HR-ESI-MS spectrum of **3**.

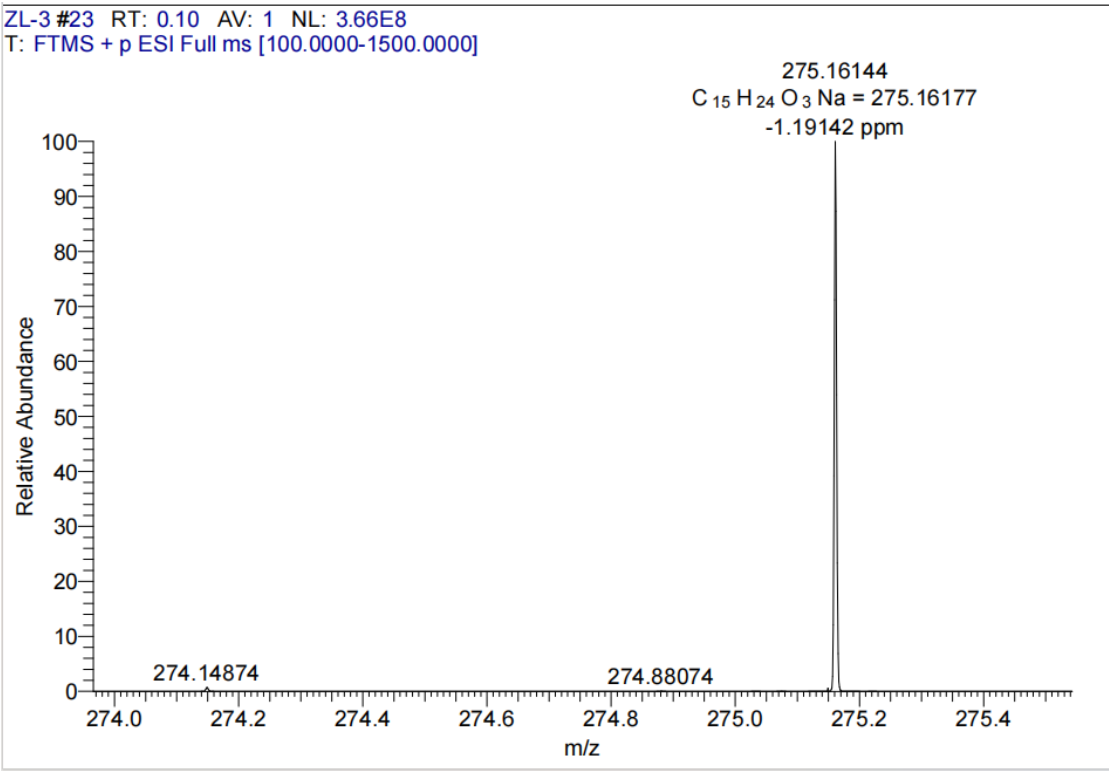

Figure S26. UV spectrum of **3**.

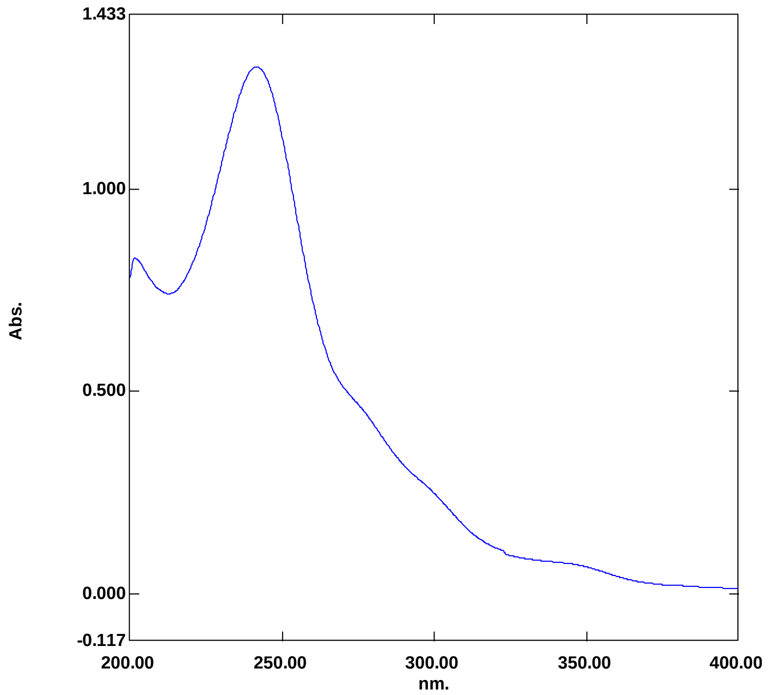

No.	P/V	Wavelength (nm)	Abs.
1		241.60	1.303
2		201.60	0.830

Figure S27. IR spectrum of **3**.

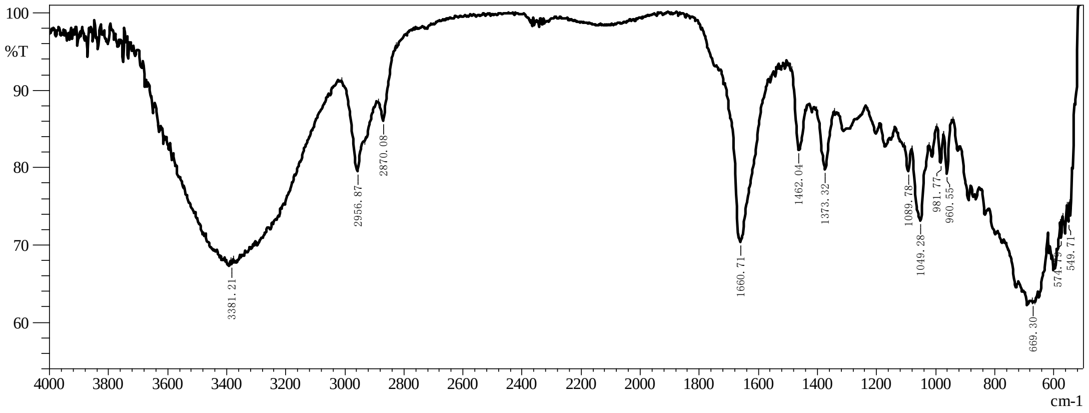

Figure S28. ^{1}H-NMR (600 MHz, CD_3OD) spectrum of **4**.

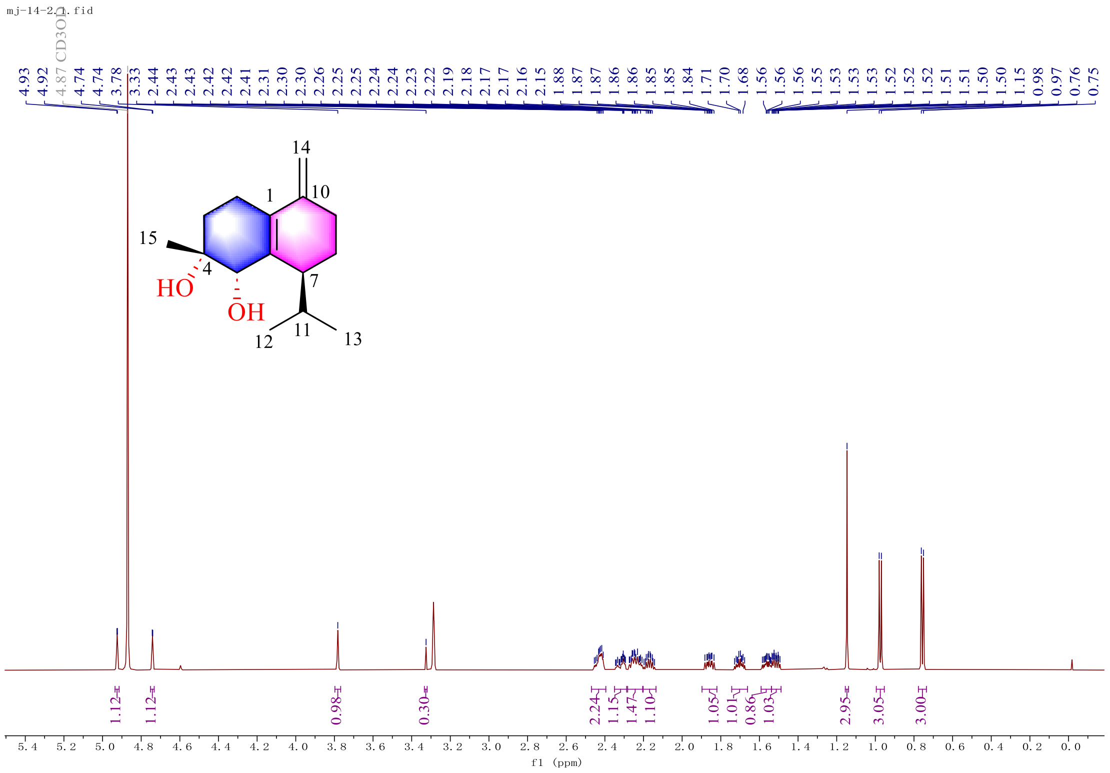

Figure S29. ^{13}C-NMR (600 MHz, CD_3OD) spectrum of **4**.

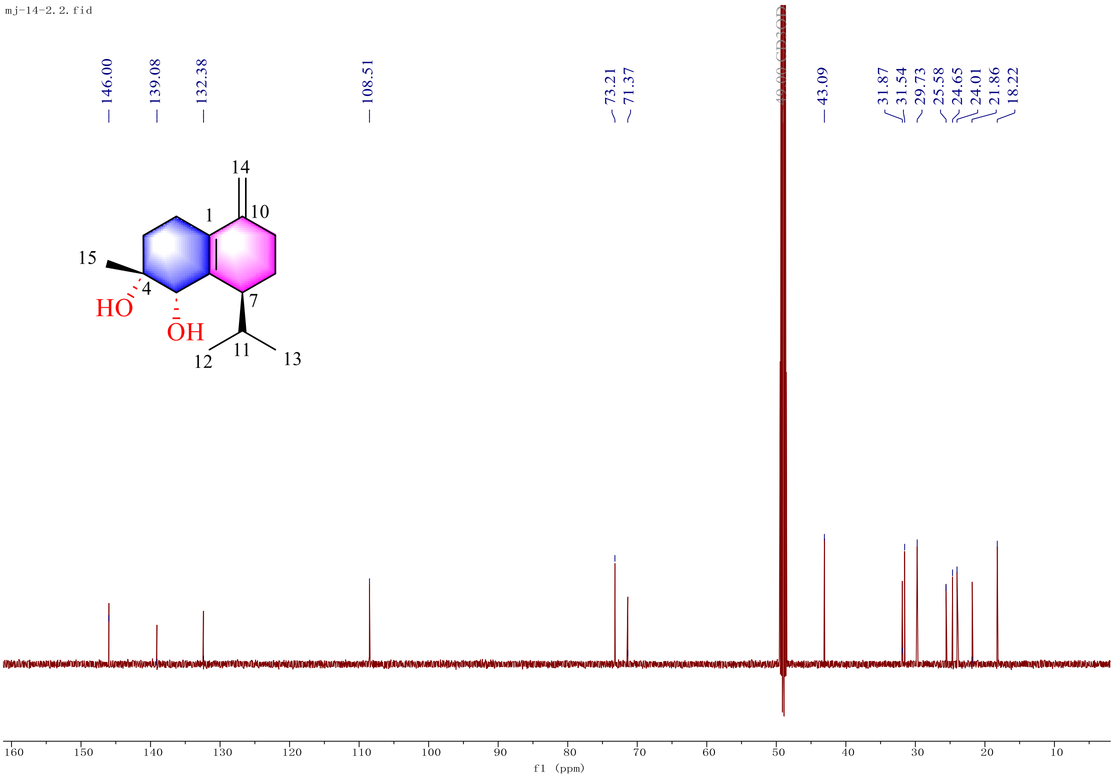

Figure S30. HSQC spectrum of **4**.

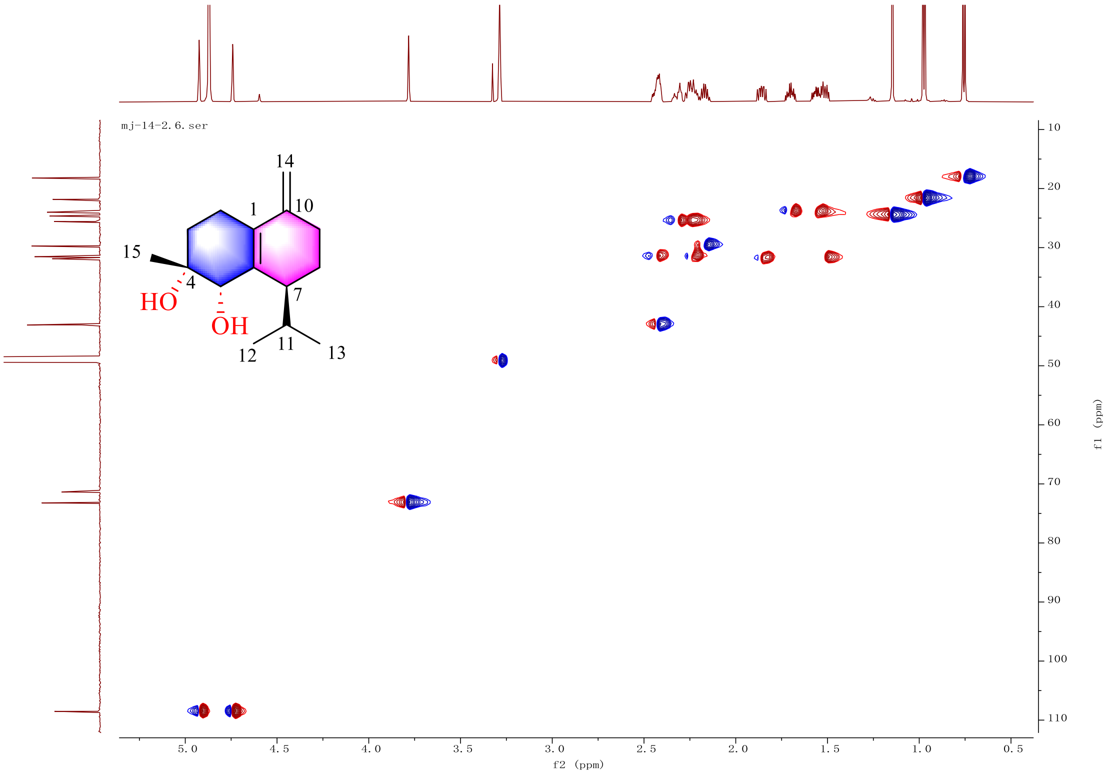

Figure S31. HMBC spectrum of **4**.

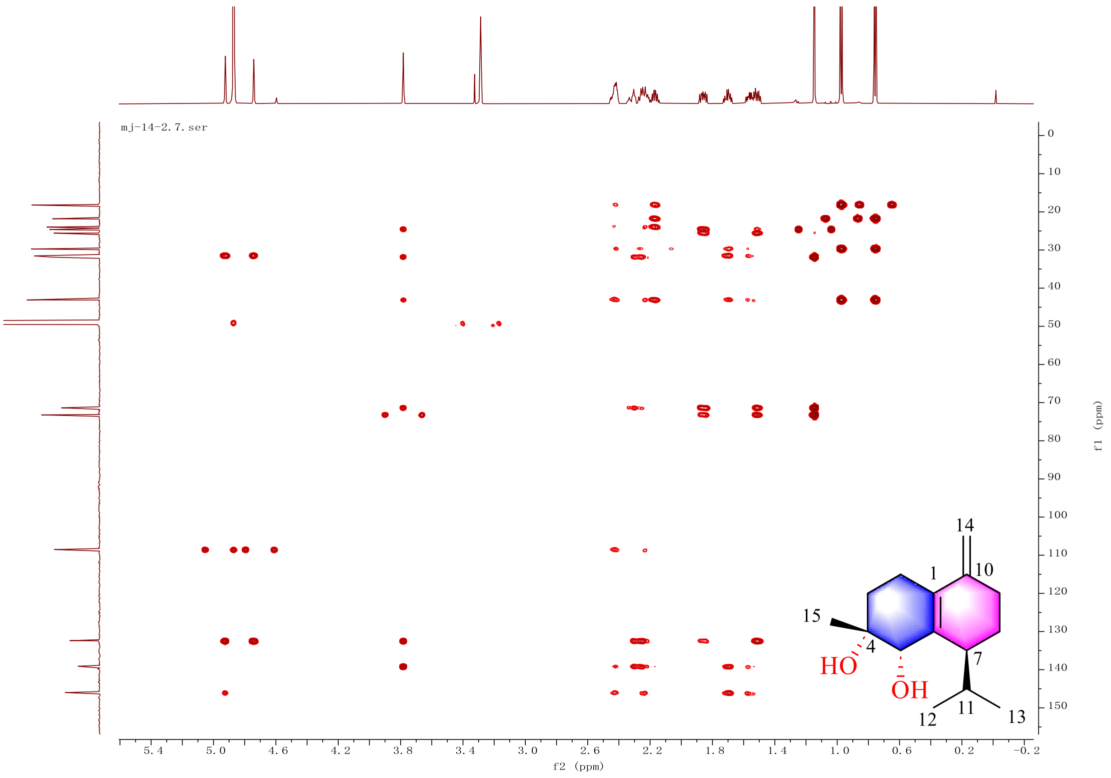

Figure S32. ^{1}H-^{1}H COSY spectrum of **4**.

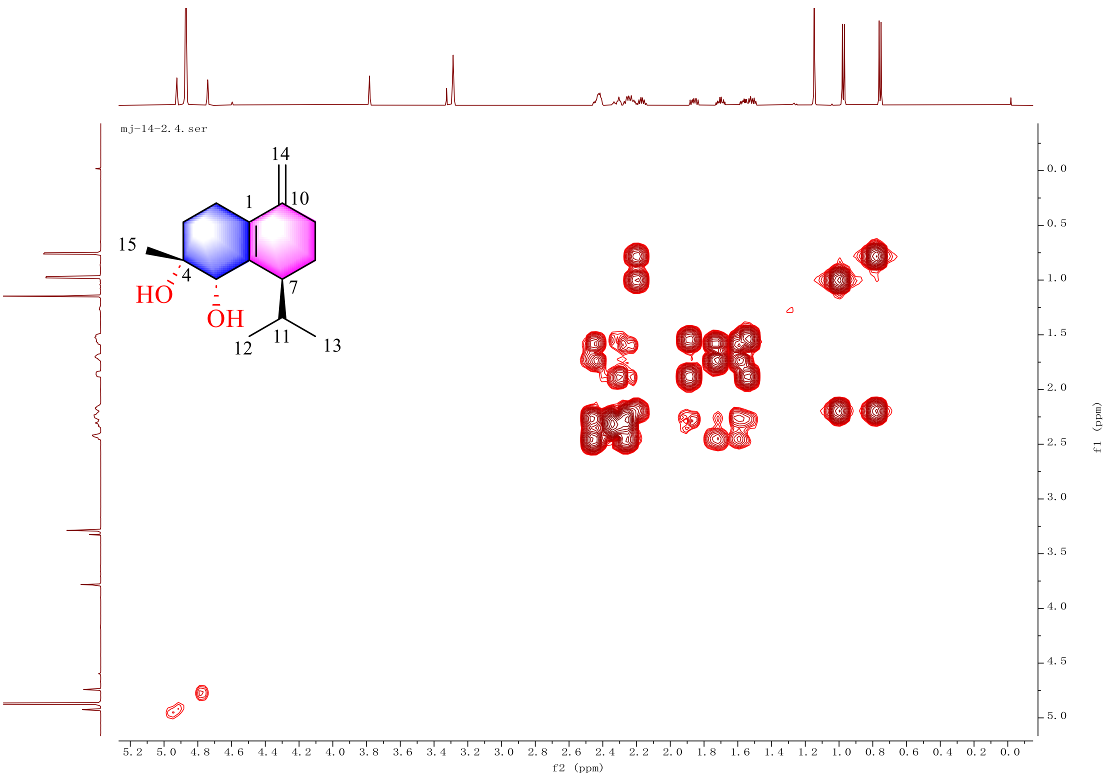

Figure S33. NOESY spectrum of **4**.

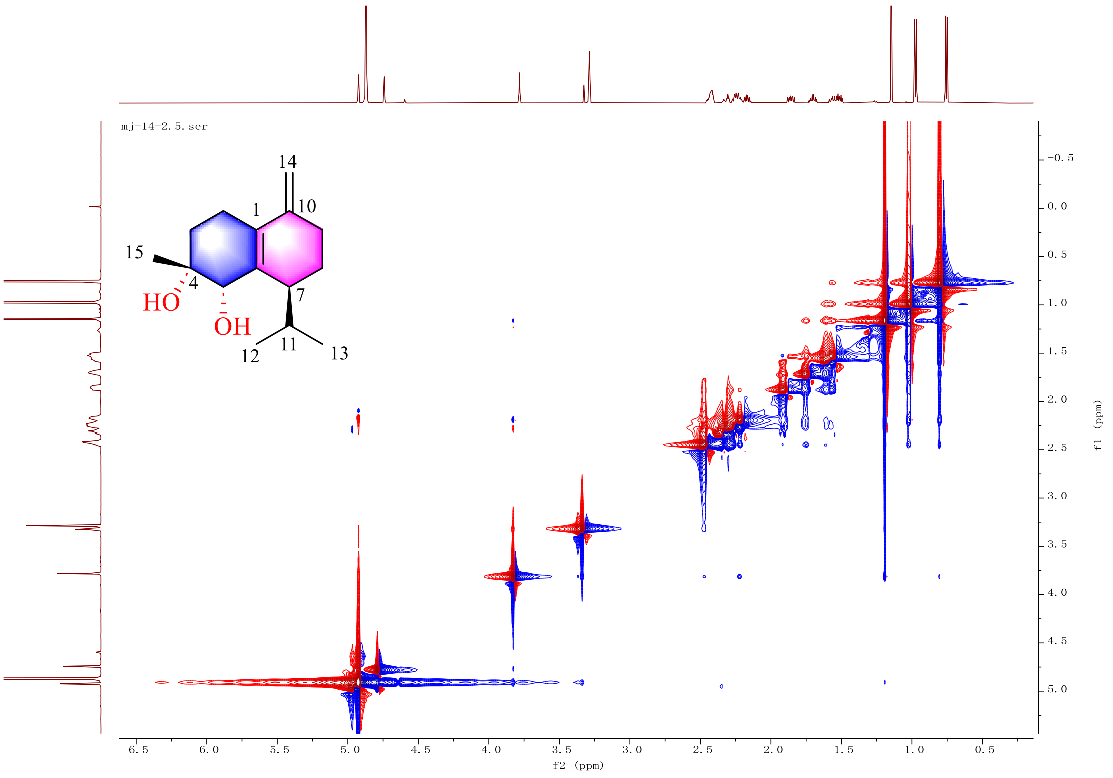

Figure S34. HR-ESI-MS spectrum of **4**.

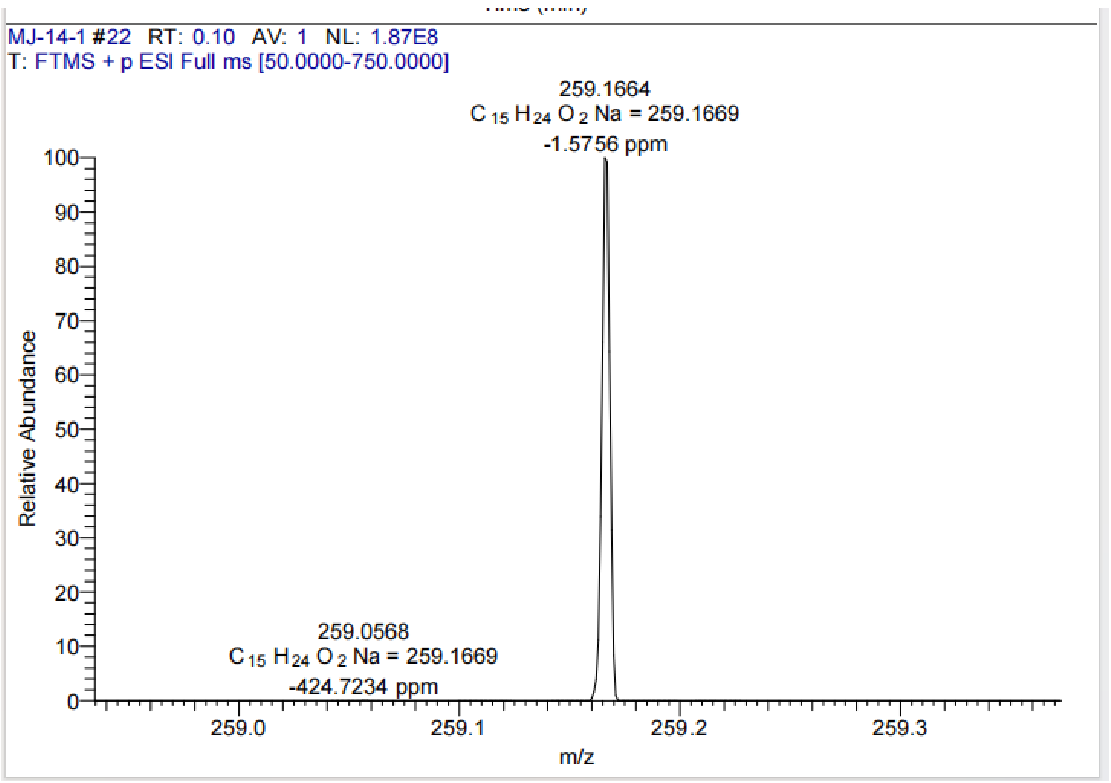

Figure S35. UV spectrum of **4**.

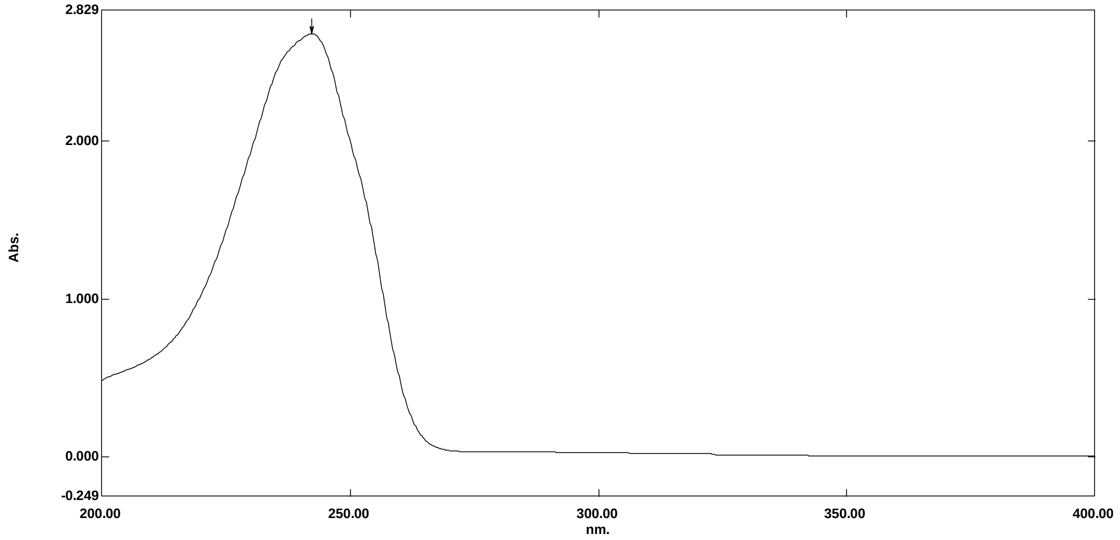

No.	Wavelength (nm)	Abs.
1	242.20	2.682

Figure S36. IR spectrum of **4**.

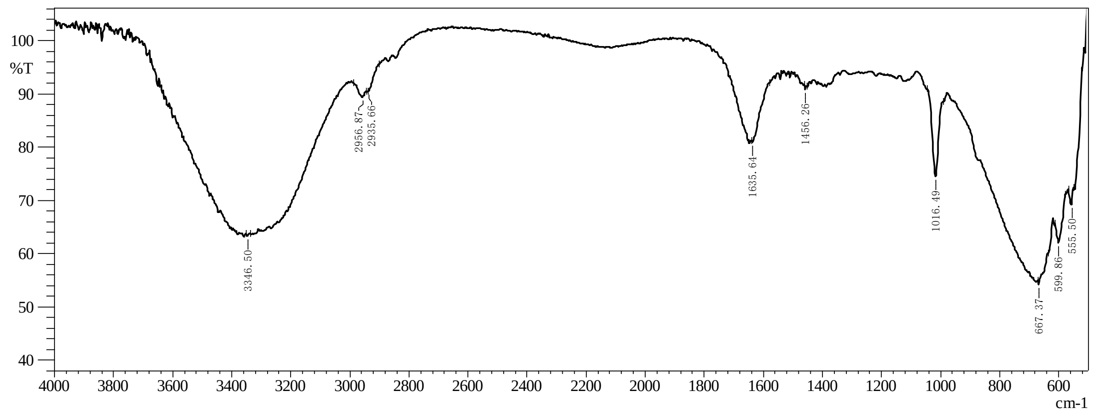

Figure S37. ^{1}H-NMR (600 MHz, CD_3OD) spectrum of **5**.

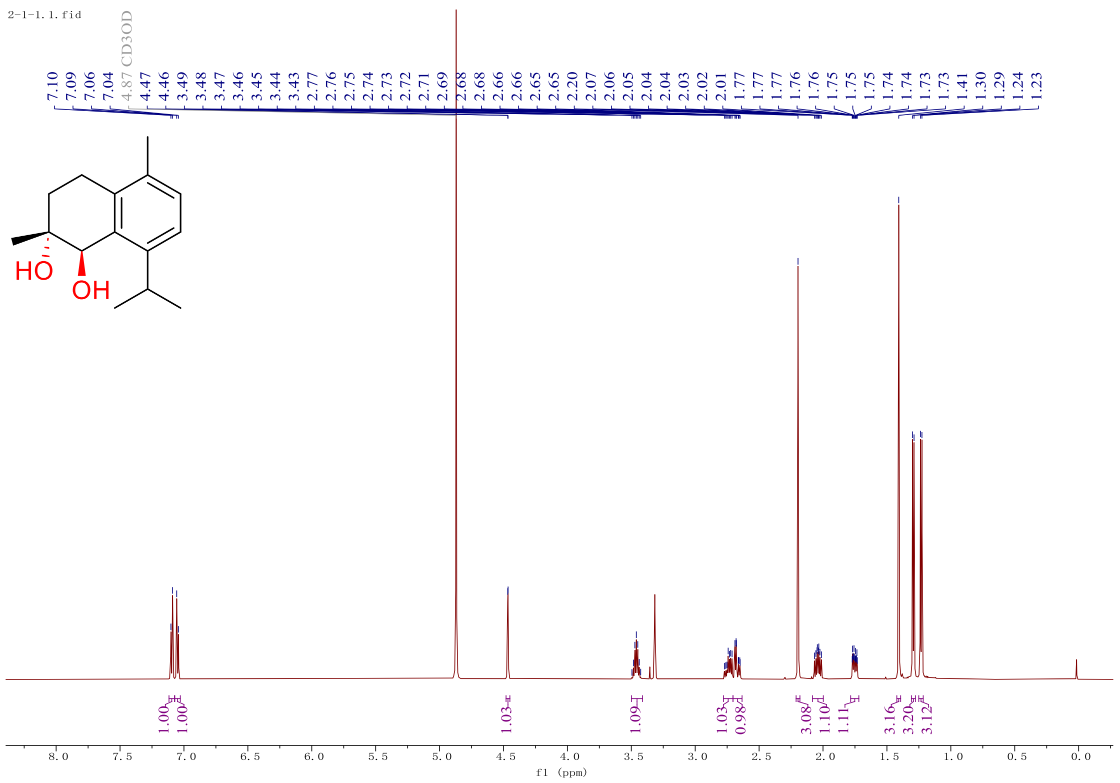

Figure S38. ^{13}C-NMR (150 MHz, CD_3OD) spectrum of **5**.

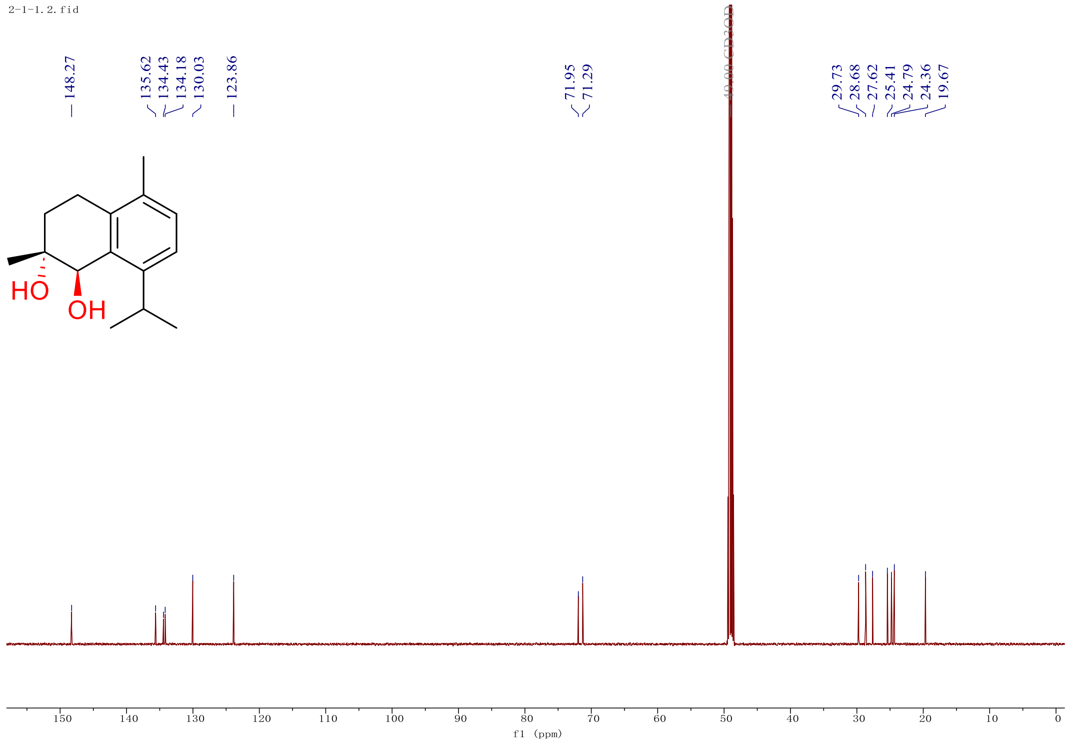

Figure S39. ^{1}H-NMR (600 MHz, CD_3OD) spectrum of **6**.

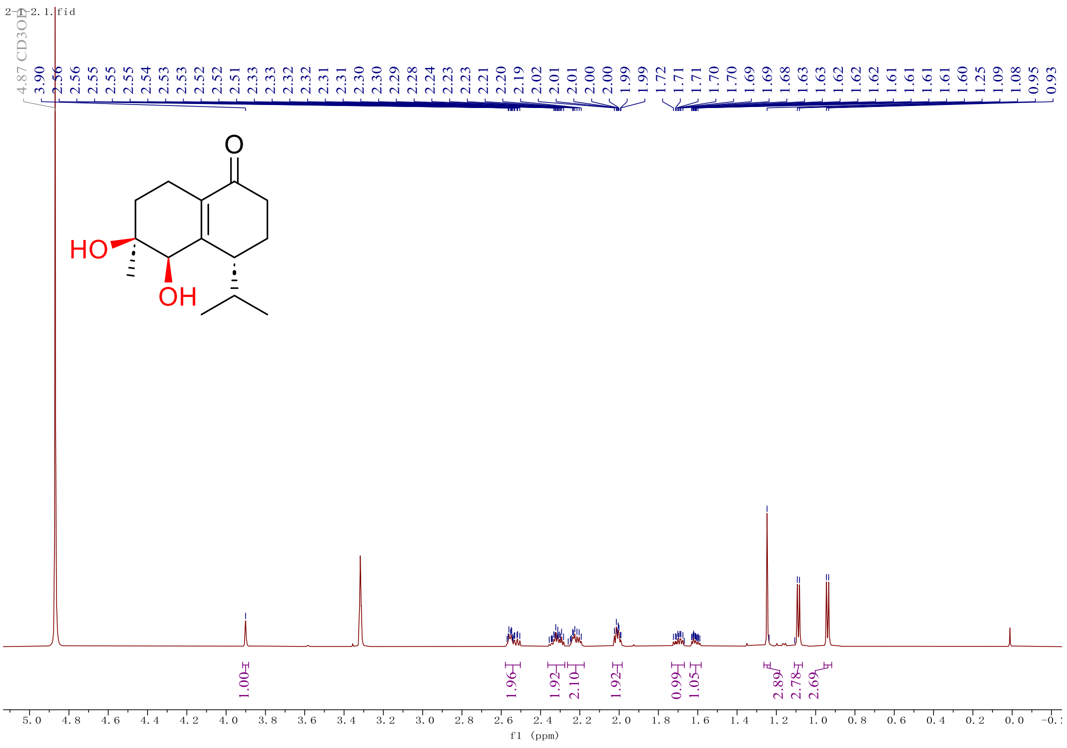

Figure S40. ^{13}C-NMR (150 MHz, CD_3OD) spectrum of **6**.

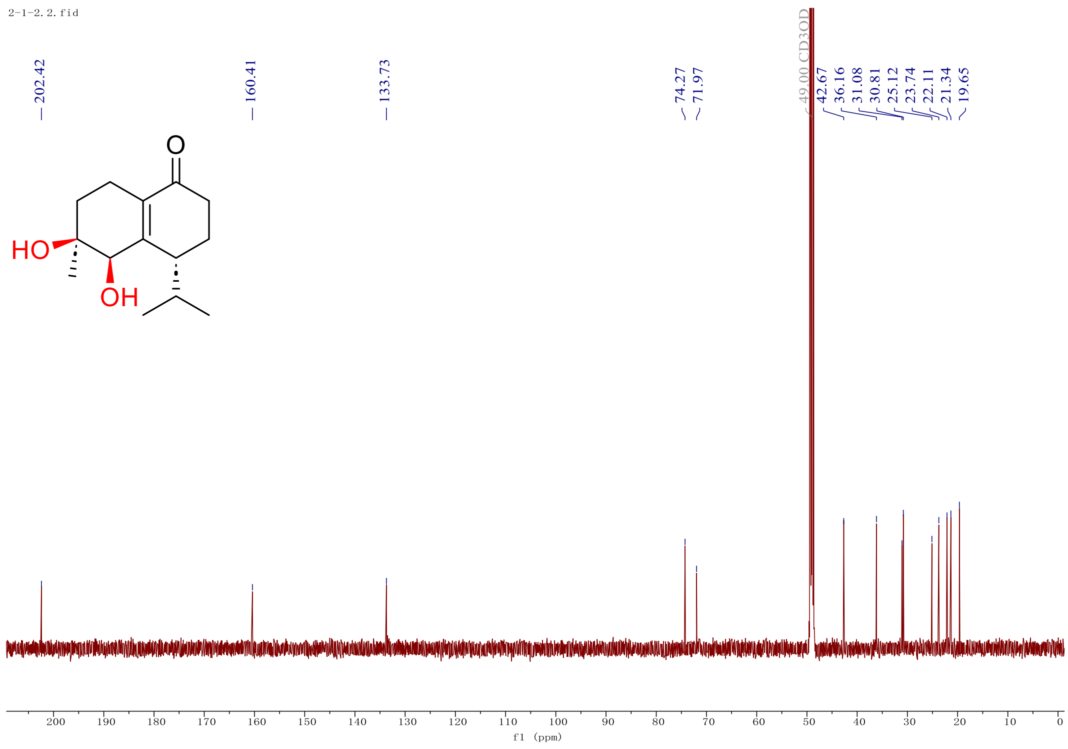

Figure S41. ^{1}H-NMR (600 MHz, CD_3OD) spectrum of **7**.

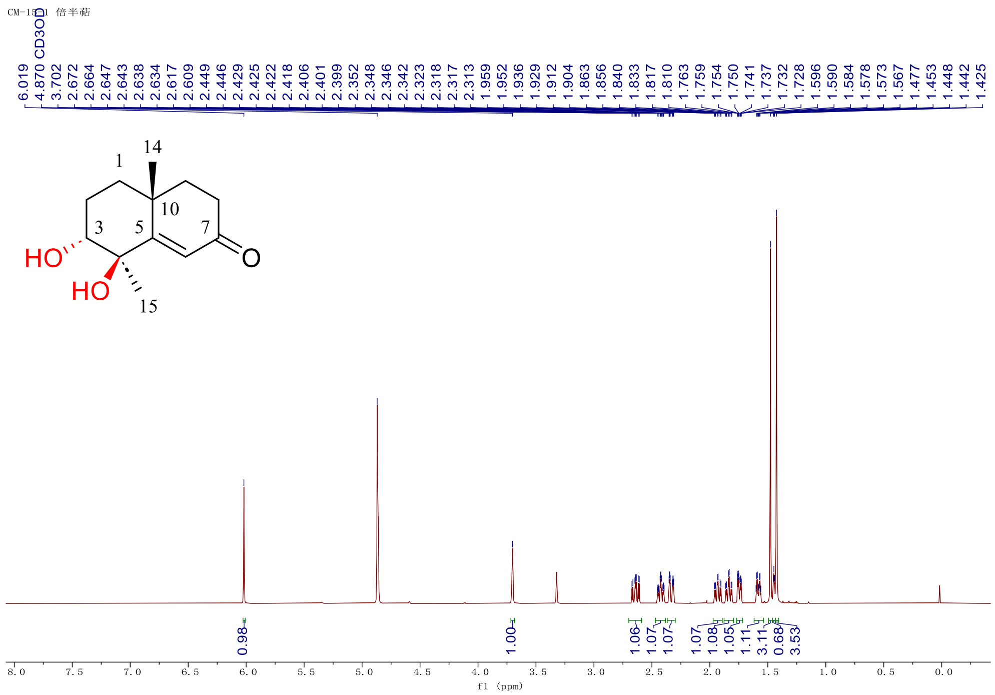

Figure S42. ^{13}C-NMR (150 MHz, CD_3OD) spectrum of **7**.

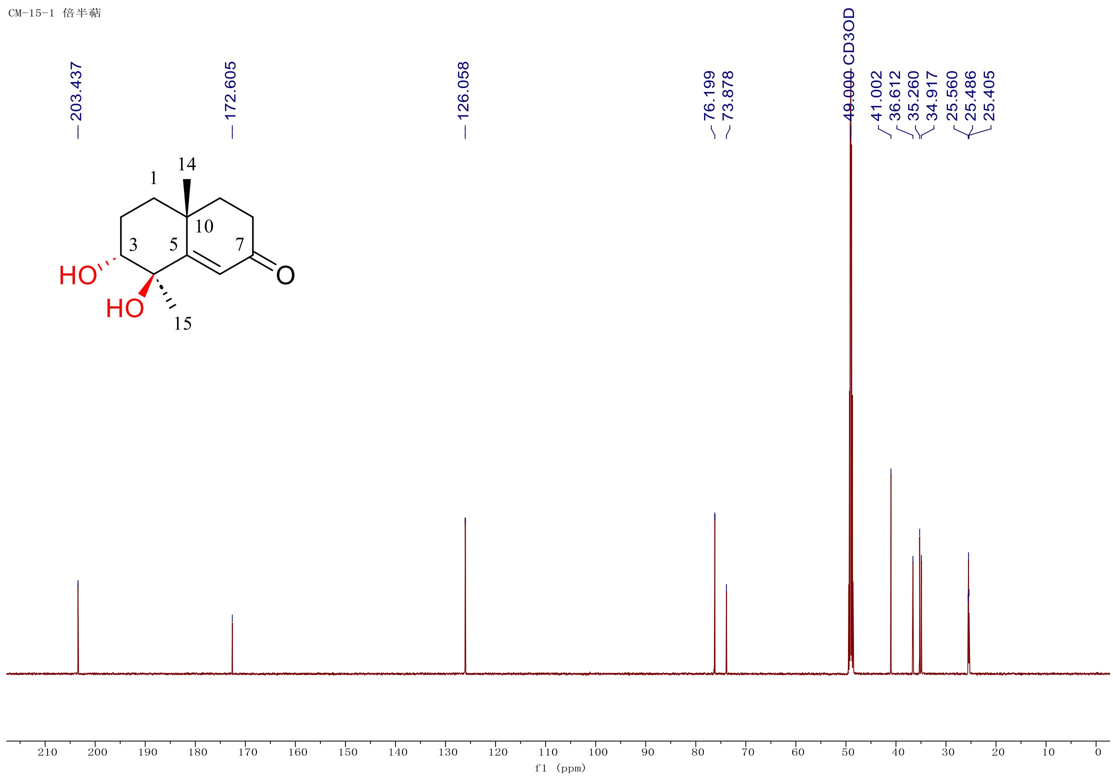

Figure S43. ^{1}H-NMR (600 MHz, $CDCl_3$) spectrum of **8**.

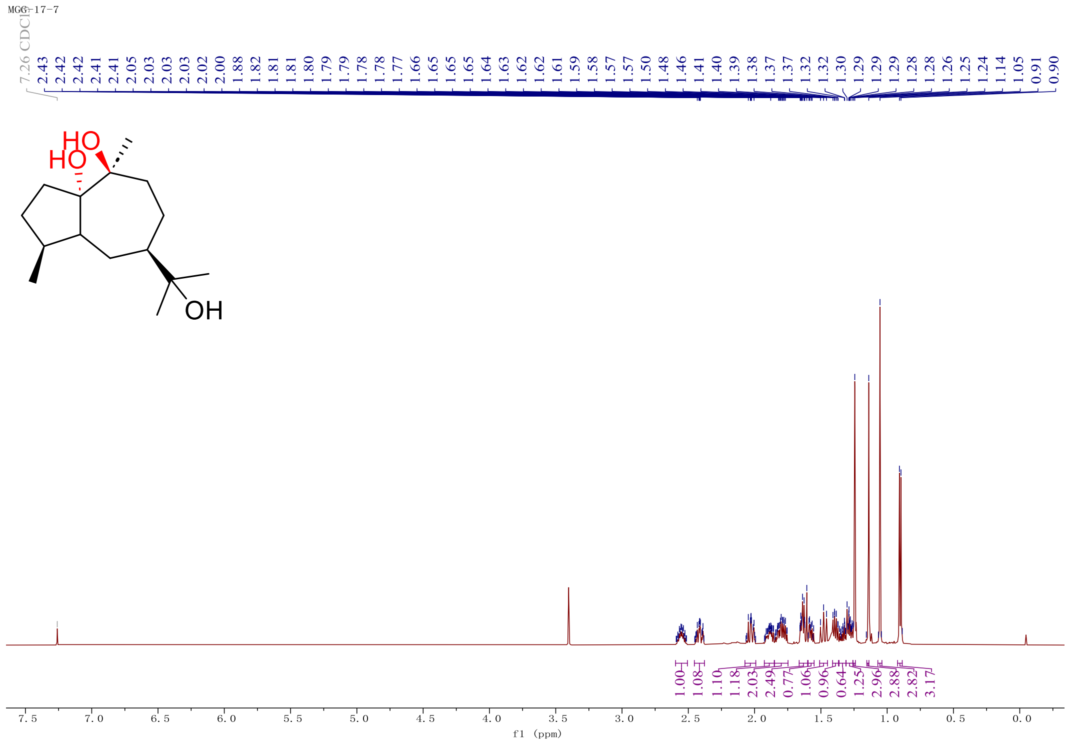

Figure S44. ^{13}C-NMR (150 MHz, $CDCl_3$) spectrum of **8**.

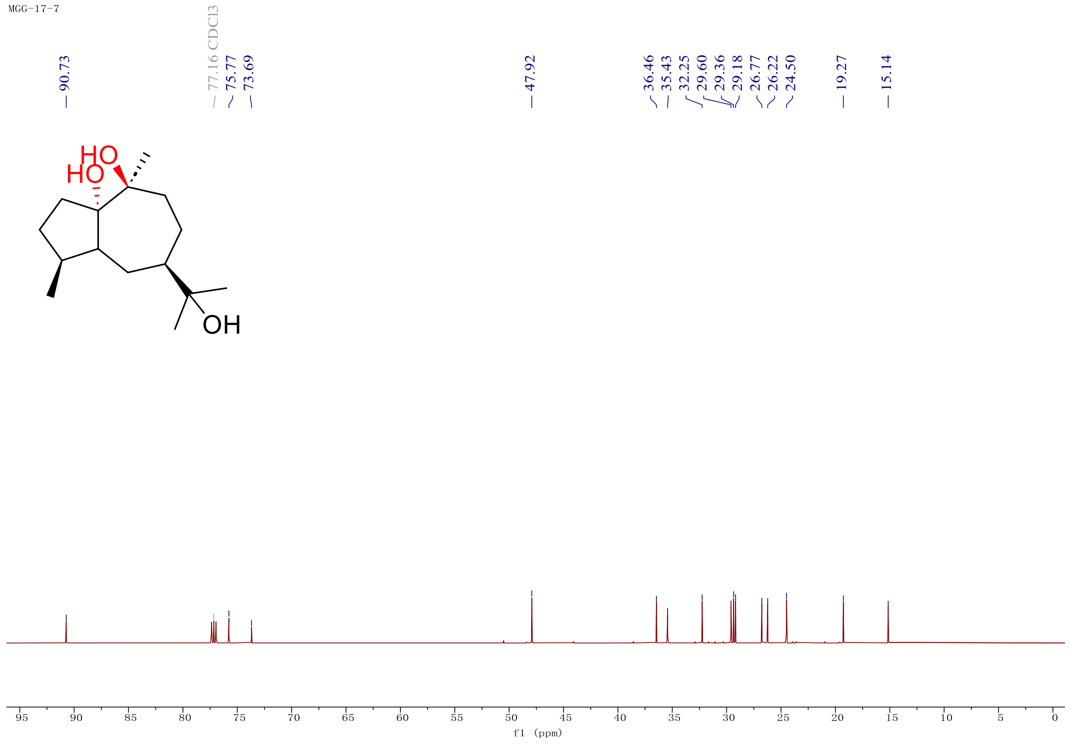

Supplement: Supplementary file 1 [file ijms-25-12693-s001.zip › ijms-3311333-supplementary.pdf]
